# Disruption of the Endothelial Barrier by Proteases from the Bacterial Pathogen *Pseudomonas aeruginosa*: Implication of Matrilysis and Receptor Cleavage

**DOI:** 10.1371/journal.pone.0075708

**Published:** 2013-09-19

**Authors:** Nathalie Beaufort, Elisabeth Corvazier, Saouda Mlanaoindrou, Sophie de Bentzmann, Dominique Pidard

**Affiliations:** 1 Inserm, U698, Paris, France; 2 Université Denis Diderot, UMR-S698, Paris, France; 3 CNRS, UMR 7255-LISM, Marseille, France; 4 Aix-Marseille University, Marseille, France; University of Miami, United States of America

## Abstract

Within the vasculature, uncontrolled pericellular proteolysis can lead to disruption of cell-to-cell and cell-to-matrix interactions and subsequent detachment-induced cell apoptosis, or anoikis, contributing to inflammatory vascular diseases, with the endothelium as the major target. Most studies so far have focused on endogenous proteinases. However, during bloodstream infections, bacterial proteinases may also trigger endothelial anoikis. We thus investigated the potential apoptotic activity of the proteinases secreted by the haematotropic opportunistic pathogen, *Pseudomonas aeruginosa*, and particularly its predominant metalloproteinase, LasB. For this, we used the secretome of the LasB-expressing pseudomonal strain, PAO1, and compared it with that from the isogenic, LasB-deficient strain (PAO1∆*lasB*), as well as with purified LasB. Secretomes were tested for apoptotic activity on cultured human endothelial cells derived from the umbilical vein or from the cerebral microvasculature. We found that the PAO1 secretome readily induced endothelial cell anoikis, as did secretomes of LasB-positive clinical pseudomonal isolates, while the PAO1∆*lasB* secretome had only a limited impact on endothelial adherence and viability. Notably, purified LasB reproduced most of the effects of the LasB-containing secretomes, and these were drastically reduced in the presence of the LasB-selective inhibitor, phosphoramidon. A precocious and extensive LasB-dependent degradation of several proteins associated with the endothelial extracellular matrix, fibronectin and von Willebrand factor, was observed by immunofluorescence and/or immunoblotting analysis of cell cultures. Moreover, the PAO1 secretome, but not that from PAO1∆*lasB*, specifically induced rapid endoproteolysis of two major interendothelial junction components, VE-cadherin and occludin, as well as of the anti-anoikis, integrin-associated urokinase receptor, uPAR. Taken as a prototype for exogenous haemorrhagic proteinases, pseudomonal LasB thus appears to induce endothelial anoikis not only *via* matrilysis, as observed for many pro-apoptotic proteinases, but also *via* cleavage of some essential cell-to-cell and cell-to-matrix adhesion receptors implicated in the maintenance of the endothelial barrier.

## Introduction

Proteinases associated with focal or systemic inflammation in the vessel wall play a prominent role in modulating permeability of the endothelial barrier, endothelial cell (EC) proliferation and migration, and the balance between EC survival and apoptosis. These include serine-proteinases of the plasma coagulation and fibrinolytic systems, or secreted by activated leukocytes, as well as metalloproteinases, either secreted or membrane-associated [1,2]. A noticeable pathogenic activity of inflammatory proteinases on vascular cells, particularly ECs, is their capacity to induce the disassembly of cell-to-cell and/or cell-to-matrix connections [3,4] that then triggers an apoptotic type of programmed cell death called anoikis [5-8].

The vascular endothelium is a major target for many human pathogenic bacteria and their virulence factors. Upon entering the bloodstream, they can dampen and escape from the immune defenses, and, in order to disseminate into the body, they alter or even disrupt the endothelial barrier, thus inducing severe pathological events within the vasculature [9-12]. The Gram-negative bacterium *Pseudomonas aeruginosa* is such an opportunistic human pathogen, and a major agent for bloodstream infections, particularly in immunocompromised patients, leading to focal and/or systemic severe vascular diseases [13-15]. This bacterium expresses numerous virulence factors targeting several cell types, including phagocytes, epithelial cells and ECs. Among these factors are several cell wall-associated adhesins and invasins, potent intracellular toxins delivered into host cells through multimolecular secretion systems, and a panel of extracellular proteinases [13,15-18]. The latter are mostly metalloproteinases or serine-proteinases, and are considered as virulence factors in various human pseudomonal infections [15,16,19]. Particular emphasis has been placed on the pseudomonal elastase, LasB/pseudolysin, a member of the thermolysin/M4 superfamily of metallopeptidases that includes proteinases secreted by several common pathogenic haematotropic bacteria [16,20]. It is the major protein(ase) secreted by *P. aeruginosa* [21], is widely expressed among pseudomonal clinical isolates, particularly those from patients with blood infections [22,23], and participates in bacterial escape from the host immune system, host colonization, and tissue destruction [16,19,24,25]. Notably, LasB can be regarded as a prototype of exogenous proteinases altering hemostasis [19,25].

The aptitude of pathogenic bacteria to alter the viability of host cells, including ECs, *via* secreted effector proteins is well established. However, investigations have mainly focused on toxins that can be transferred into the cytoplasm of target cells, as shown for *P. aeruginosa* [15,17]. The possible role of bacterial extracellular proteinases in such a process, in addition to host proteinases, remains under assessment, particularly for ECs [26,27]. Thus, despite the propensity of *P. aeruginosa* to produce severe infections within the vasculature and its capacity to induce programmed cell death of cultured ECs [17,28], the impact of secreted pseudomonal proteinases on EC survival has so far been little investigated [25]. In the present study, we thus examined the impact of secreted pseudomonal proteinases with cultured human ECs of various vascular origins. Extending our previous observations made on human vascular mesenchymal cells [29] to barrier-forming cells such as ECs, we now show that, among pseudomonal exoproducts, the metalloproteinase LasB is largely responsible for the induction of EC detachment and death (anoikis), *via* both matrilysis and receptor proteolysis. Thus, in addition to proteolysis of fibronectin (Fn), LasB readily degrades the EC matrix-specific protein von Willebrand factor (vWf). Furthermore, LasB specifically and directly degrades interendothelial junctional proteins such as VE-cadherin and occludin, as well as uPAR, an important integrin-associated membrane protein involved in cell adhesion to matrix and cell survival, all these proteolytic events being thus likely to participate in endothelial anoikis.

## Materials and Methods

### Reagents

Rabbit polyclonal and mouse monoclonal antibodies (pAb and mAb, respectively) were from the following sources: anti-Fn pAb F3648, from Sigma-Aldrich (Saint-Louis, MO); anti-vWf pAb A0082, from DakoCytomation (Glostrup, Denmark); anti-glyceraldehyde-3-phosphate dehydrogenase (GAPDH) mAb 2D4A7, from Abcam (Cambridge, UK); anti-VE-cadherin pAb BMS158, from BenderMedSystems (Vienna, Austria); anti-occludin pAb 71-1500, and anti-claudin-5 pAb 34-1600, from Invitrogen Corp. (Camarillo, CA); anti-uPAR domain 2 mAb #3932, from American Diagnostica (Greenwich, CT), or a gift from Dr. Viktor Magdolen (Technische Universität Munchen, Germany). Control antibodies were mAb MOPC-21 and rabbit polyclonal serum IgGs, from Sigma-Aldrich and DakoCytomation, respectively, while horseradish peroxidase-, or Alexa Fluor 555-conjugated IgGs were from Jackson Immunoresearch (West Grove, PA) and Invitrogen, respectively.

Proteinase inhibitors phosphoramidon (PA; from *Streptomyces* *tanashiensis* ) and aprotinin (Apro; from bovine lung) were from Sigma-Aldrich, while batimastat (BB-94) was from BioVision Research Products (Mountain View, CA). Polymyxin B sulfate (PMB; from *Bacillus* *polymyxa* ) was from Sigma-Aldrich.

Purified human vWf (Laboratoire français du Fractionnement et des Biotechnologies, Les Ulis, France) was stored at -20°C at 1 mg/ml (≈ 3.6 μM based on a vWf monomer molecular mass ≈ 280 kDa). Purified LasB (specific activity, 260 U/mg; Elastin Products Company, Owensville, MO) was kept at -80°C at 0.5 mg/ml (≈ 15 μM based on a molecular mass ≈ 33 kDa). Its purity and enzymatic activity have been previously described [29].

### Preparation of extracellular bacterial secretomes and proteinase activity assays

The previously characterized LasB-producing *P. aeruginosa* strain PAO1 and its LasB-deficient isogenic mutant PAO1∆*lasB* [30,31] were mostly used in this study. Preparation of sterile bacterial secretomes (hereafter abbreviated as PAO1-Sec and PAO1∆*lasB*-Sec, respectively) in Luria-Bertani broth (LB) liquid medium (Invitrogen) was performed exactly as previously described [21,29], and is detailed in Protocol S1 in File S1. Psa1-Sec and Psa2-Sec are two secretomes that have been fully described previously, and were prepared with LasB-producing, *P. aeruginosa* clinical isolates derived from patients with infective endocarditis subsequent to implantation of a pacemaker [29]. Analysis of proteinases in bacterial secretomes by gelatin zymography and Elastin-Congo red hydrolysis assay was performed as reported [21,29,32,33], and is detailed in Protocol S1 in File S1. Experimental dilutions of secretomes or LB control medium are expressed as the percentage of bacterial culture supernatant in the final reaction volume.

### Cell culture

Batches of human umbilical vein endothelial cells (HUVECs) from single donors at first passage were obtained from Promocell Bioscience (Heidelberg, Gemany), as were cell culture medium (Endothelial Cell Basal Medium) and supplements. Cells were seeded on plastic dishes (TPP, Trasadingen, Swiss) precoated with 0.1 mg/mL purified rat tail collagen type I (Coll.I ; BD Biosciences, San Jose, CA), grown to confluence, and passaged after brief trypsinization in the continuous presence of 5 U/ml penicillin, 0.5 μg/ml streptomycin, and 25 ng/ml amphotericin B (PSA; Gibco-Invitrogen). Experiments were performed on cell cultures between second and fifth passage.

The human adult brain microvascular endothelial cell line hCMEC/D3 is an immortalized cell line that was established through lentiviral transduction of hTERT and SV40 large T antigen into a primary culture of adult brain endothelial cells [34], and was provided by Dr. Pierre-Olivier Couraud (Institut Cochin, Paris, France). Culture medium was Endothelial Basal Medium 2 (EBM-2; Lonza, Basel, Swiss) supplemented with 5% (v/v) fetal calf serum (FCS) (Eurobio, Les Ulis, France), 1 ng/mL basic fibroblast growth factor, 5 mg/mL ascorbic acid, 1.4 mM hydrocortisone (all from Sigma-Aldrich), and 10 mM 4-(2-hydroxyethyl)-1-piperazineethanesulfonic acid (HEPES), containing PSA and 1000 U/mL plasmocin (Invitrogen). hCMEC/D3 cells were seeded on Coll.I-coated plastic dishes, and passaged at confluence *via* trypsinization. Experiments were performed on cultures at passages 31 to 35.

### Exposure of proteins or endothelial cells to pseudomonal secretomes or proteinases

Purified vWf was adjusted to 5 μg/mL (≈ 20 nM) in Tris 50 mM, NaCl 200 mM, pH 7.4 (Tris/NaCl) containing 0.2% FCS, and 100 ng fractions were exposed for 24 h at 37°C to purified LasB (100 nM), to bacterial secretomes or to LB medium in the range of 1 to 10%, in a final volume of 20 μl. The reaction was stopped by addition of sodium dodecyl sulfate (SDS) and heating, before SDS-polyacrylamide gel electrophoresis (SDS-PAGE) coupled to immunoblotting (IB) analysis.

Confluent human EC cultures were rinsed with culture medium containing low (i.e. 0.2%) FCS, then cells were exposed to either LB medium or bacterial secretomes in the range of 1 to 20%, to purified LasB in the range of 50 to 200 nM, or to 5 μM staurosporine (Stauro; Sigma-Aldrich), all diluted in low FCS culture medium for a final volume of ≥ 0.15 ml/cm^2^, and maintained at 37°C for variable times. Cells were observed by phase contrast light microscopy and photographed at the end of treatment. In some experiments, incubation of cells was performed in parallel in the presence of the proteinase inhibitors PA (50 μM), BB-94 (40 nM), or Apro (10 μM), or of the antibiotic PMB (20 μg/mL).

### Collection of cell culture treatment supernatants and preparation of cell extracts

Following exposure of cell cultures to tested substances, treatment media were collected, and detached floating cells were pelleted by centrifugation at 2,000*g* for 5 min at 20°C. Cell-free media were further centrifuged at 18,000*g* for 30 min at 4°C to remove debris, and supernatant then stored at -80°C until analysis. Residual adherent cells were lyzed for 30 min on ice with a radioimmunoprecipitation assay (RIPA) medium containing a cocktail of proteinase inhibitors (P8340; Sigma-Aldrich), and cell lysates were processed as previously detailed [29] for further analysis.

### Cell assays

Cell quantity assay using Crystal violet (CV; Sigma-Aldrich) staining, cell viability assay using tetrazolium dye (MTT; Sigma-Aldrich) metabolism, cell death assays using either the terminal deoxynucleotidyl transferase dUTP nick end labeling (TUNEL; *In Situ* Cell Death Detection kit; Roche Applied Science, Mannheim, Germany) and cell nucleus counterstaining with 4',6-diamidino-2-phenylindole (DAPI; Sigma-Aldrich), or Trypan blue exclusion, or total cell protein measurement (BCA kit; Pierce, Stonehouse, UK) were all performed exactly as previously described [29] on residual adherent cells and/or floating cells immediately following exposure of EC cultures to tested substances.

### Immunoblot analysis

Proteins separated by SDS-PAGE were immunoblotted exactly as previously described [21,29], and as detailed in the figure legends. Films were scanned with a GS-800 Calibrated Densitometer (Bio-Rad) and images were treated with the PDQuest software (Bio-Rad). Bands corresponding to molecular species of interest were quantified through densitometric plot using the NIH ImageJ v1.46r software.

### Immunofluorescence microscopy analysis

HUVECs or hCMEC/D3 cells grown in Coll.I-coated Permanox-type Lab-Tek slides (Nalge Nunc International, Rochester, NY) were exposed to tested substances, and residual adherent cells were fixed with 3.7% (w/v) paraformaldehyde. Immunostaining of ECM proteins or cell receptors was performed as reported [29], and as detailed in the figure legends. Immunolabeling was followed by sequential labeling of cell F-actin with Alexa Fluor 488-coupled phalloidin (Invitrogen), and staining of cell nuclei with DAPI. Cells were finally prepared for fluorescent microscopy, and images were recorded and prepared with the Archimed software (Microvision Instruments, Évry, France).

### Statistics

Unless otherwise stated, quantitative data are presented as means ± standard error of the mean (SEM) of the indicated number of independent determinations, and were statistically analyzed by the unpaired, 2-tailed Student’s *t* test. *P* values are provided in the figure legends.

## Results

### The pseudomonal metalloproteinase LasB induces EC detachment and death

Investigation of the impact of secreted pseudomonal proteinases on endothelial cells was conducted on two types of cultured human ECs. The microvascular cell line, hCMEC/D3 [34], represents a pertinent EC type when studying endothelium-pathogen interactions [10,12], but may present some resistance to apoptosis due to its immortalized nature [35]. Results obtained with the microvascular cells have been thus validated by using primary ECs of venous origin, HUVECs. Confluent cell cultures were exposed to the secretome (PAO1-Sec) of the pseudomonal strain PAO1, to that (PAO1∆*lasB*-Sec) of its isogenic, experimentally-derived LasB-deficient strain or to purified LasB. As previously reported [29,33], secretomes obtained from PAO1 contain all major extracellular pseudomonal proteinases, i.e. the metalloproteinases AprA (alkaline protease/aeruginolysin) and LasB, and the serine-proteinase PrpL (protease IV) [16], as shown by gelatin zymography analysis of PAO1-Sec preparations used in this study (Figure S1 in File S1, right-hand panel). LasB concentrations in these preparations were estimated to be in the range of 250 to 300 nM, using an elastin-Congo red elastolytic assay [29] with purified LasB as reference activity (Figure S1 in File S1, left-hand panel). In contrast, PAO1∆*lasB*-Sec is totally devoid of LasB, although still containing active AprA and PrpL (Figure S1 in File S1, right-hand panel) [29].

**Figure 1:**
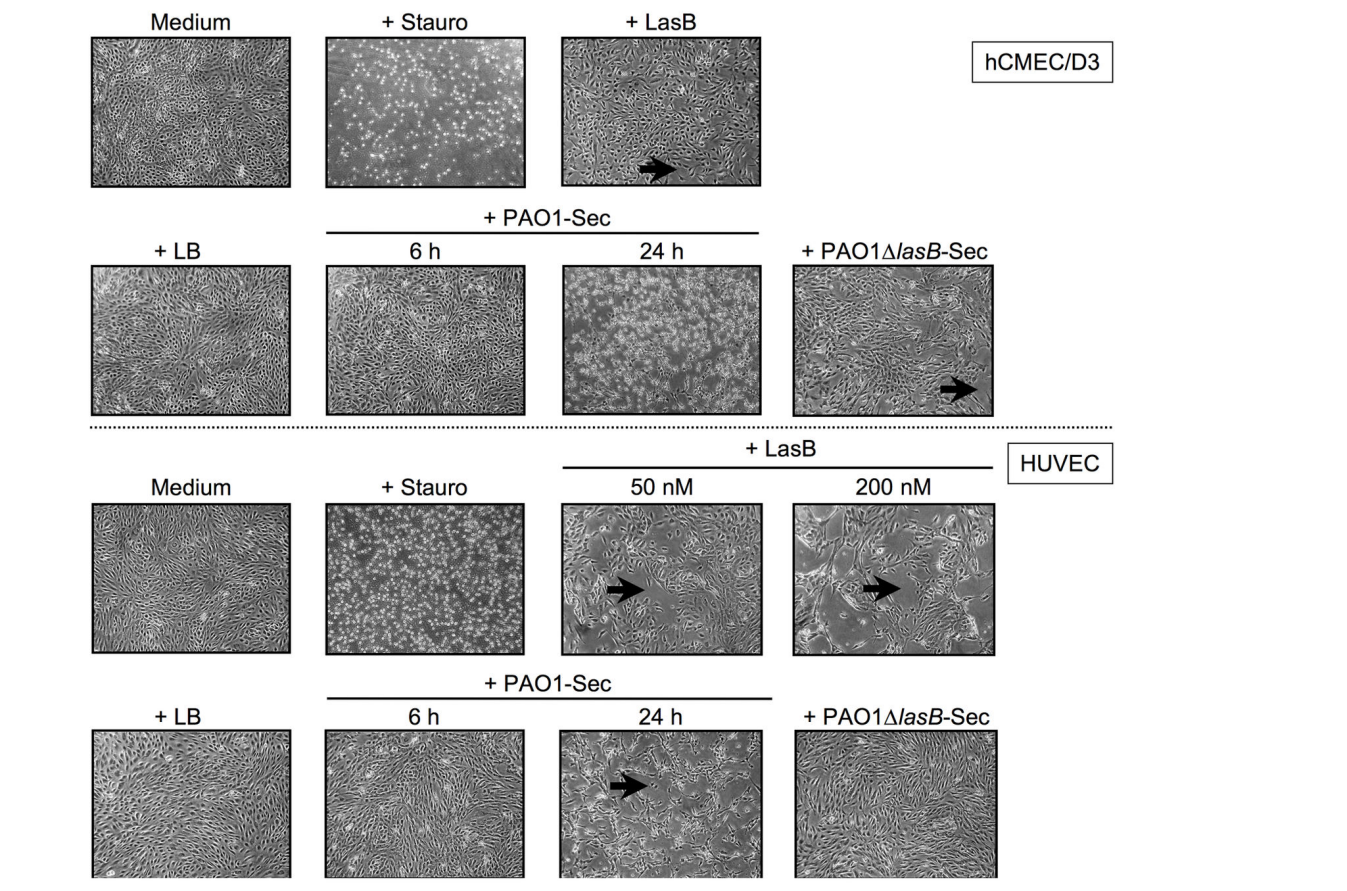
The pseudomonal secretome containing LasB and purified LasB both alter confluent human endothelial cells in culture. Confluent cultures of hCMEC/D3 cells (two upper rows) or HUVECs (two lower rows) were shifted to low (0.2% v/v) FCS culture medium before being exposed at 37°C to either medium alone as control (24 h) or supplemented with staurosporine (*Stauro*; 5 μM, 6 h) or with purified LasB (50 or 200 nM, 24 h), or with medium supplemented with 10% (v/v) LB as control (24 h), or with 10% PAO1-Sec (6 or 24 h) or 10% PAO1∆*lasB*-Sec (24 h). A representative field of the cell layers was photographed under a light microscope (40x magnification). Black arrows indicate areas of cell retraction. Images are representative of at least three similar independent experiments performed on each cell type.

When added to confluent hCMEC/D3 cells, PAO1-Sec at 10% (v/v), corresponding to LasB concentrations in the range of 25 to 30 nM, produced little morphological changes of cells within 6 h, but cell retraction, rounding and detachment became evident at 24 h (Figure 1, upper panels). The control LB medium showed no effect on cell density or morphology, whereas the pro-apoptotic agent staurosporine produced a massive cell rounding and detachment within only a few hours (Figure 1, upper panels), as expected [36]. Similar observations were made on HUVECs (Figure 1, lower panels). Remarkably, when exposed to 50 nM purified LasB, cultures showed morphological changes, with cells becoming elongated and cultures exhibiting areas of cell retraction (Figure 1, upper and lower panels), while increasing LasB to 200 nM resulted in large acellular areas, as depicted in Figure 1 (lower panels) for HUVECs. In sharp contrast, 10% PAO1∆*lasB*-Sec had little effect, with only cell elongation, loosening of cell-to-cell connections and discrete areas of cell retraction to be seen at 24 h of exposure (Figure 1, upper and lower panels).

**Figure 2:**
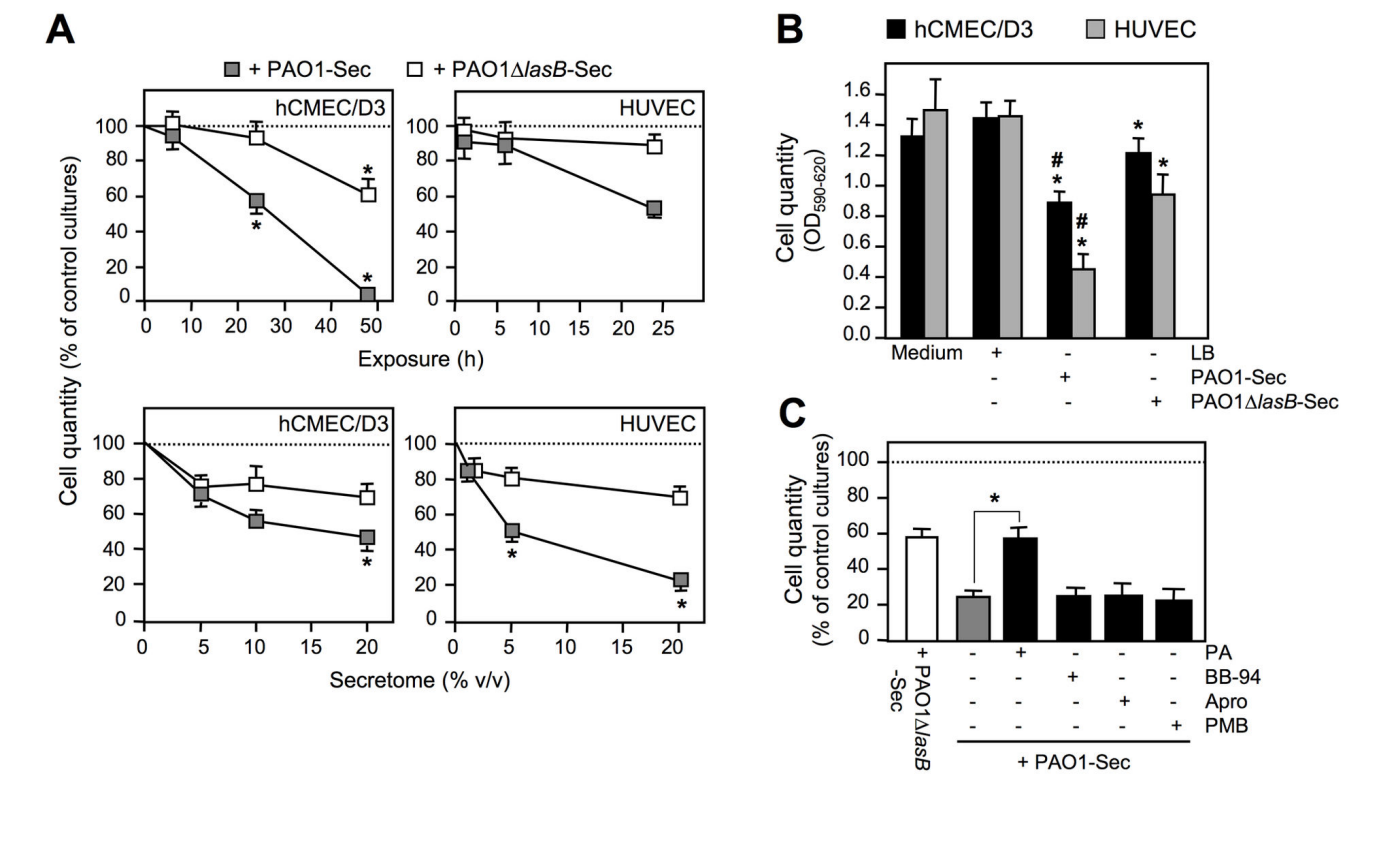
The pseudomonal secretome containing LasB drastically reduces adherence of human endothelial cells in culture. (*A*) Residual adherent cells in confluent cultures exposed for 6 to 48 h (hCMEC/D3 cells) or 1 to 24 h (HUVECs) to 10% PAO1-Sec or PAO1∆*lasB*-Sec (upper panels), or exposed for 24 h to 5 to 20% (hCMEC/D3 cells) or 1 to 20% (HUVECs) PAO1-Sec or PAO1∆*lasB*-Sec (lower panels) were quantified using the CV staining assay. Results are expressed as percentages of the mean OD value measured at 590 nm on triplicate wells after subtraction of the value at 620 nm for control LB-treated cultures, and are reported as the mean ± SEM of three independent experiments, except for HUVECs exposed to secretomes for increasing periods of time (mean and range of two independent experiments). * *P* < 0.05 *versus* cell cultures exposed to control LB medium. (*B*) Quantification of residual adherent cells was performed using the CV staining assay in EC cultures exposed for 24 h to low FCS culture medium alone or with 10% of either LB, PAO1-Sec, or PAO1∆*lasB*-Sec, reported as OD values, means + SEM of five (HUVECs) to six (hCMEC/D3 cells) independent experiments. * *P* ≤ 0.05 *versus* culture medium alone or with LB; # *P* < 0.05 *versus* medium with PAO1∆*lasB*-Sec. (*C*) Quantification of residual adherent cells was performed using the CV staining assay for HUVEC cultures exposed for 24 h to low FCS culture medium with 10% of either LB (control cultures), PAO1-Sec, or PAO1∆*lasB*-Sec, in the absence (grey bar) or presence (black bars) of either phosphoramidon (50 μM; *PA*), batimastat (40 nM; *BB-94*), aprotinin (10 μM; *Apro*), or polymyxin B (20 μg/mL; *PMB*). Results are reported as in (A) for 3 independent experiments. * *P* < 0.05.

Upon exposure of cultures to the bacterial secretomes, residual adherent cells were quantified using the Crystal violet colorimetric assay, confirming that, on a time course basis, the number of adherent ECs, both hCMEC/D3 cells and HUVECs, sharply decreased beyond 6 h of exposure to 10% PAO1-Sec, being reduced, at 24 h, by ≈ 50% as compared to control culture wells (Figure 2A, upper panels). No cell remained adherent at 48 h (Figure 2A, upper hCMEC/D3 panel). Varying the amount of bacterial secretome in the range of 1 to 20% over a 24 h period showed that a significant loss of adherent cells occurred for PAO1-Sec ≥ 5% (corresponding to ≥ 12.5 nM LasB), with maximal effects seen for 10 to 20%, with HUVECs appearing slightly more susceptible to detachment than hCMEC/D3 cells (Figure 2A, lower panels). Indeed, taking an exposure of confluent ECs to 10% PAO1-Sec for 24 h as the optimal condition to evaluate EC detachment, residual adherent hCMEC/D3 cells and HUVECs appeared to be significantly reduced by ≈ 35% and ≈ 70%, respectively, as compared to control cultures (Figure 2B). Furthermore, exposure of the hCMEC/D3 cell cultures to 50 nM purified LasB for 24 h resulted in a ≈ 40% reduction in adherent cells (data not illustrated). In contrast, PAO1∆*lasB*-Sec had quantitatively a most limited impact on EC adherence. Although inducing a statistically significant cell loss when compared to control LB-treated cultures (*P* ≤ .05) for exposures of 24 h or longer, cell detachment remained significantly lower compared to that induced by PAO1-Sec (*P* ≤ .02) (Figure 2A-B). When exposure of EC cultures to PAO1-Sec was performed in the presence of various proteinase inhibitors, inclusion of phosphoramidon (PA), an inhibitor specific for LasB among pseudomonal proteinases [20,29], was found to reduce the loss of adherent HUVECs produced by PAO1-Sec to the level seen with PAO1∆*lasB*-Sec (Figure 2C). By contrast, neither aprotinin, a potent inhibitor of plasmin [37], nor BB-94, a wide-spectrum inhibitor of MMPs [38], had any protective effect against the PAO1-Sec-induced loss of adherent HUVECs (Figure 2C). Finally, cell cultures were also exposed to PAO1-Sec in the presence of polymyxin B, an inhibitor of bacterial wall-derived LPS [39], which was unable to counteract the detachment of HUVECs (Figure 2C). Similar results were obtained with hCMEC/D3 cells exposed to 10% PAO1-Sec or PAO1∆*lasB*-Sec, with or without PA (data not illustrated), as well as with HUVECs exposed to 5% Psa1-Sec or Psa2-Sec, two secretomes derived from clinical *P. aeruginosa* blood isolates that contained ≈ 450 nM LasB [29], with or without PA (Figure S2 in File S1). Taken together, these observations indicated that LasB plays a prominent role in the massive alteration of EC adherence induced by proteinase-producing pseudomonal strains.

**Figure 3:**
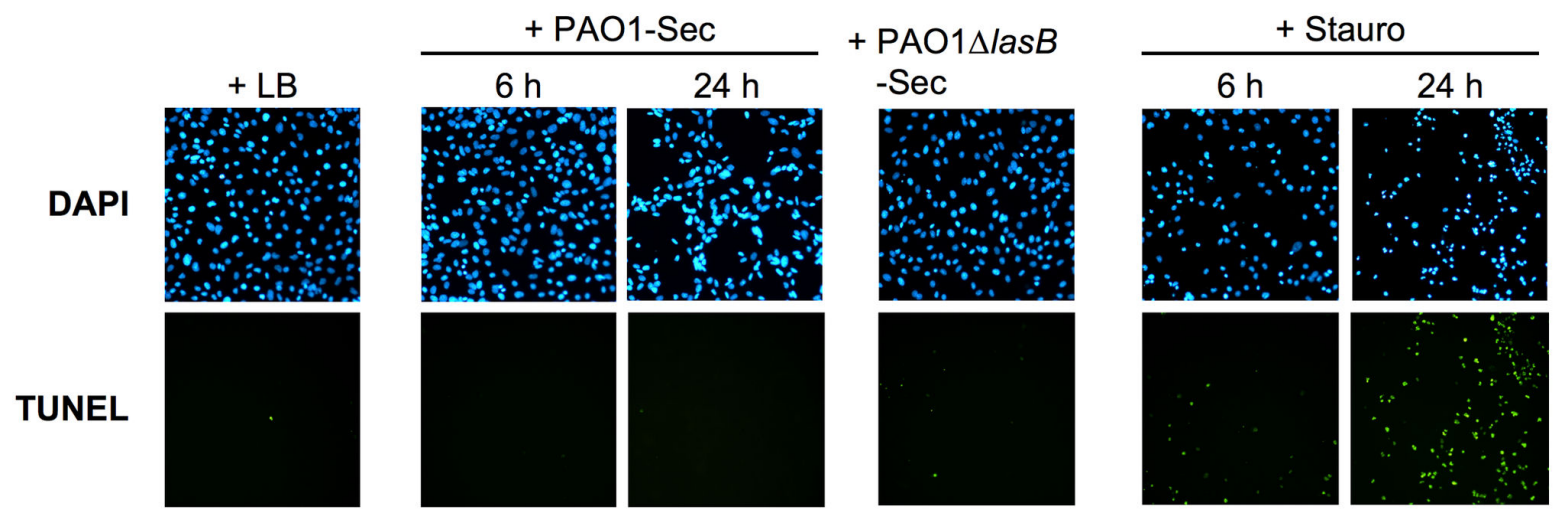
Residual adherent endothelial cells in the presence of pseudomonal secretomes are not apoptotic. Confluent hCMEC/D3 cell cultures were exposed for up to 24 h in low FCS culture medium with 10% of either LB, PAO1-Sec, or PAO1∆*lasB*-Sec, or to staurosporine. Residual adherent cells were evaluated for apoptotic nuclei by staining with DAPI (upper panels) or the TUNEL assay (lower panels). Representative areas (100x magnification) are shown for one of three similar experiments performed on either hCMEC/D3 cell or HUVEC cultures. Cultures with LB or PAO1∆*lasB*-Sec are depicted at 24 h.

Remarkably, the adherent ECs remaining after exposure to PAO1-Sec were viable, showing no evidence of an apoptotic phenotype. Indeed, after exposure to 10% PAO1-Sec for 6 or 24 h, or to PAO1∆*lasB*-Sec for 24 h, residual adherent ECs displayed DAPI-stained nuclei with normal appearance, as compared with control LB-treated cells (Figure 3, upper panels), while only rare, if any, cells positive for TUNEL were observed, indicating fragmented DNA (Figure 3, lower panels). In contrast, cells exposed to 5 μM staurosporine showed pycnotic and TUNEL-positive nuclei after only 6 h (Figure 3, upper and lower panels, respectively), a feature that applied to all residual adherent cells by 24 h. Viability of residual adherent cells was confirmed by measuring in parallel total cells *via* CV staining and viable cells *via* MTT metabolism, and by calculating the MTT/CV ratio of cultures as an index of their viability. Although exposure of both types of ECs to 5 or 20% of PAO1-Sec for 24 h resulted in a marked loss of adherent cells, the MTT/CV ratio remained statistically comparable between PAO1-Sec-treated, PAO1∆*lasB*-Sec-treated, and control LB-treated cultures, while staurosporine markedly altered this ratio (Figure S3 in File S1). Conversely, ≈ 80% of the floating cells recovered from cultures after 48 h with PAO1-Sec were Trypan blue-positive dead cells, whereas the MTT assay indicated no metabolic activity (data not illustrated). These observations thus clearly indicate a process of anoikis, i.e. cell detachment followed by death, in EC cultures exposed to LasB-containing pseudomonal secretomes.

### Pseudomonal LasB cleaves ECM proteins involved in the adherence of ECs

**Figure 4:**
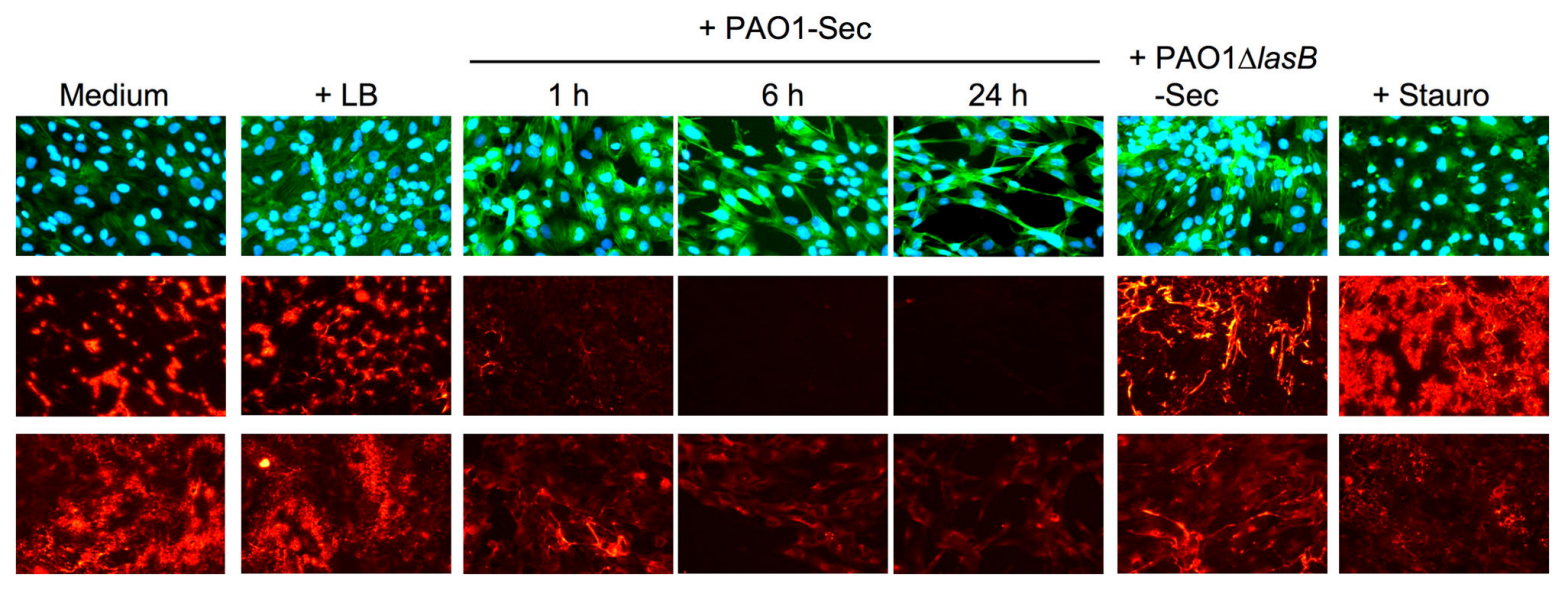
The pseudomonal LasB metalloproteinase induces matrilysis in human endothelial cell cultures. Confluent hCMEC/D3 cell cultures were exposed for 1 to 24 h to low FCS culture medium alone or with 10% LB, PAO1-Sec, or PAO1∆*lasB*-Sec, or for 4 h to staurosporine. Cell cultures were then fixed and observed by (immuno) fluorescence microscopy following labeling with primary pAbs against Fn (1 μg/ml, middle panels) or vWf (2.5 μg/ml, lower panels) that were revealed with a secondary Ab coupled to Alexa Fluor 555 (orange signals), then sequential labeling of the cell cytoskeleton with Alexa Fluor 488-coupled phalloidin and of nuclei with DAPI (upper panels, green and blue signals, respectively). Representative areas (400x magnification) are shown for one of three similar experiments performed on either hCMEC/D3 cell or HUVEC cultures. Cultures with LB or PAO1∆*lasB*-Sec are depicted at 24 h. Upper and middle panels show the same area of the same culture chamber for cell detection or ECM-associated Fn labeling, respectively, while lower panels show areas in another set of chambers with the same culture similarly treated for labeling of vWf.

Anoikis of vascular cells induced by extracellular proteinases can involve degradation of ECM components [6,7]. The subendothelial ECM is essentially composed of collagens, Fn, vitronectin (Vn), and von Willebrand factor (vWf) [40-43], and all these adhesive proteins are involved to some degree in EC survival [4,5,35,44]. Using immunofluorescence (IF) microscopic analysis to evaluate the integrity of ECM in EC cultures when exposed to bacterial secretomes, we first showed that exposure to 10% PAO1-Sec resulted in a rapid decrease in immunolabeling of ECM-associated Fn, already markedly evident after 1 h (Figure 4, middle panels), similarly to what we have recently reported for mesenchymal vascular cells [29]. By contrast, EC cultures exposed for 24 h to PAO1∆*lasB*-Sec showed no major change in the overall Fn immunolabeling intensity as compared to control cultures. Staurosporine-treated EC cultures displayed an apparent increase in immunolabeling of ECM-associated Fn (Figure 4, middle panels), likely due to an increased accessibility of ECM proteins to antibodies following cell rounding. vWf is another important cell adhesive component of the subendothelial ECM, that is secreted by ECs as highly multimerized homopolymers [41-43]. Immunodetection of vWf on fixed, nonpermeabilized control EC cultures gave a diffuse labeling pattern, combining an intense pericellular labeling, as previously observed [43], with a low-intensity intracellular, perinuclear labeling (Figure 4, lower panels), likely due to partial membrane permeabilization following fixation. Addition of 10% PAO1-Sec to EC cultures resulted, as early as 1 h later, in a drastic reduction in immunolabeling of ECM-associated vWf, that was almost abrogated at 6 h, leaving only the intracellular labeling (Figure 4, lower panels). In contrast, EC cultures exposed for 24 h to PAO1∆*lasB*-Sec showed no prominent reduction in the immunolabeling of ECM-associated vWf, as was also the case with staurosporine (Figure 4, lower panels).

**Figure 5:**
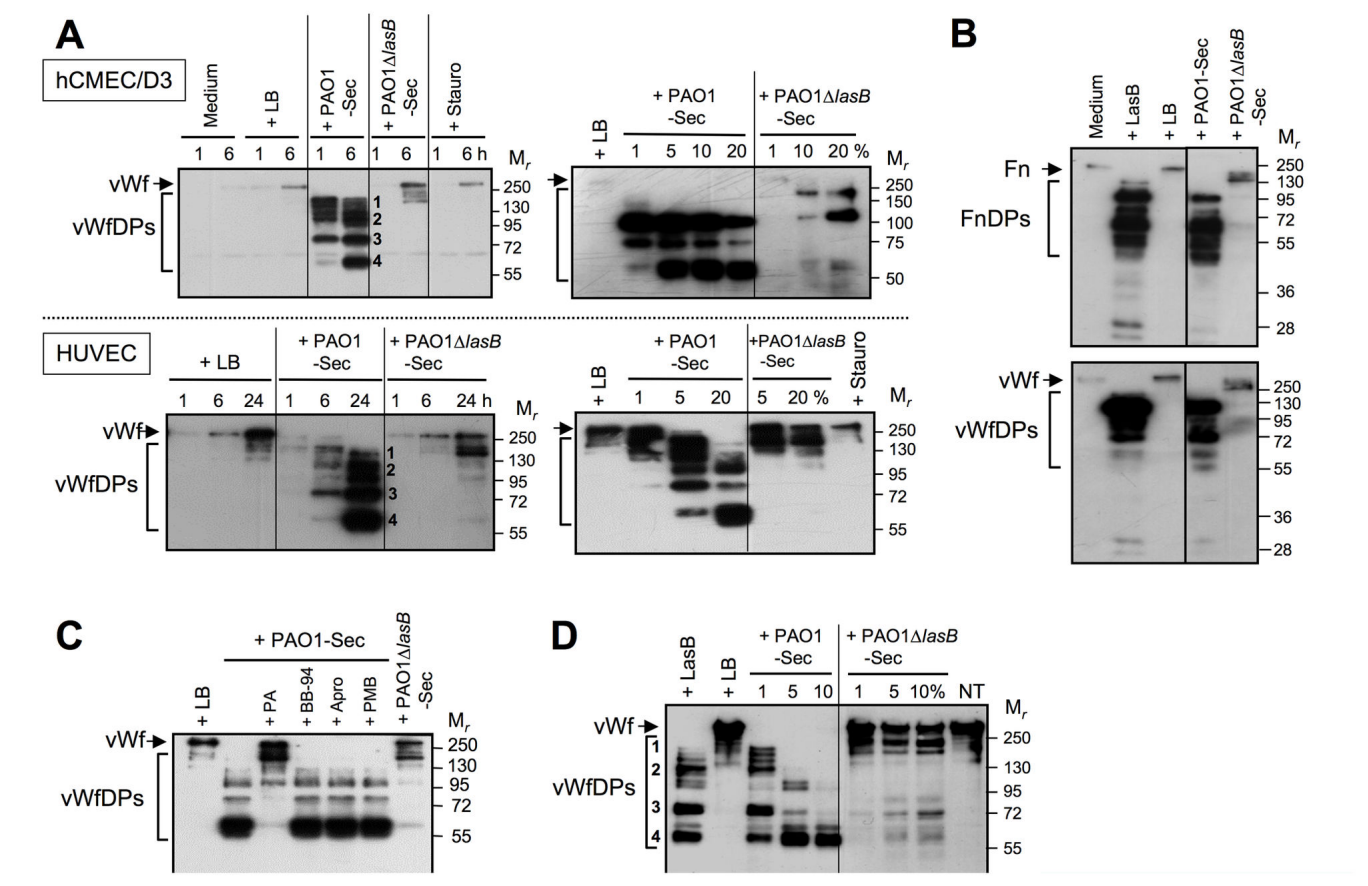
The pseudomonal LasB metalloproteinase directly targets several adhesive proteins in human endothelial cell cultures. (*A*) Confluent EC cultures were exposed for 1 to 24 h to low FCS culture medium alone or with 10% of either LB, PAO1-Sec, or PAO1∆*lasB*-Sec, or with staurosporine (left-hand panels), or for 24 h to culture medium with LB (20%) or staurosporine, or with the bacterial secretomes in the range of 1 to 20% (right-hand panels). Extracellular culture supernatants were collected and SDS-PAGE/IB was applied to disulfide-reduced proteins (20 μL supernatant *per* well), for analysis of soluble vWf degradation products (vWfDPs) using an anti-vWf pAb (0.3 μg/ml). Results illustrated are representative of three independent experiments, and major vWfDP species seen with PAO1-Sec are numbered 1 to 4. (*B*) Confluent hCMEC/D3 cell cultures were exposed for 24 h to low FCS culture medium alone, with 50 nM purified LasB, or with 10% of either LB, PAO1-Sec, or PAO1∆*lasB*-Sec. Extracellular culture supernatants were analyzed by SDS-PAGE/IB for soluble Fn degradation products (*FnDPs*, upper panel) using an anti-Fn pAb (1 μg/ml), or for vWfDPs (lower panel). (*C*) Confluent HUVEC cultures were exposed for 24 h to low FCS culture medium with 10% LB, or with 10% PAO1-Sec with or without either one of the proteinase inhibitor PA, BB-94, or Apro, or the LPS antagonist PMB, as indicated in the legend to Figure 3, or with 10% PAO1∆*lasB*-Sec. Then extracellular culture media were collected and prepared for SDS-PAGE/IB analysis of vWfDPs, as described above. Major vWfDP species seen with PAO1-Sec and with purified LasB are numbered 1 to 4. (*D*) Purified human vWf (0.1 µg) was incubated for 24 h at 37°C in 20 µL of Tris/NaCl, pH 7.4, alone (nontreated, *NT*), with 100 nM purified LasB, or with 10% LB or PAO1-Sec or PAO1∆*lasB*-Sec in the range of 1 to 10%, then proteins were analyzed by SDS-PAGE/ for vWfDPs. Results illustrated are representative of three similar experiments.

The search for soluble degradation products in culture supernatants using SDS-PAGE/IB confirmed the marked matrilysis induced by LasB. As previously reported for human vascular mesenchymal cells [29], large amounts of Fn degradation products (FnDPs) were produced during exposure of EC cultures to PAO1-Sec, whereas PAO1∆*lasB*-Sec resulted in a very limited degradation of ECM-associated Fn, quantitatively and qualitatively (Figure 5B, upper panel). For what is concerned with the endothelial matrix vWf, limited amounts of the intact protein could be detected over a 1 to 24 h period in media of EC cultures with medium with or without LB (Figure 5A, left-hand panels), appearing essentially as the full-length monomer with M_*r*_ ≈ 256 kDa [42]. This contrasted with the marked amounts of vWf degradation products (vWfDPs) that accumulated in the supernatant of cell cultures exposed to 10% PAO1-Sec for 1 to 24 h (Figure 5A, left-hand panels). Four major vWfDPs could be detected (noted 1 to 4 in Figure 5A), of mean M_*r*_ ≈ 191, 113, 79 and 64 kDa, respectively, with a shift from larger to shorter species with increasing time of exposure to PAO1-Sec. Actually, vWfDPs could be detected in the supernatant of EC cultures exposed to as little as 1% (hCMEC/D3 cells) or 5% (HUVECs) of PAO1-Sec for 24 h (Figure 5A, right-hand panels), that is, about 2.5 to 5 nM LasB. In contrast, PAO1∆*lasB*-Sec resulted in a very limited production of vWfDPs, that were only detected after 24 h of cell exposure to PAO1∆*lasB*-Sec ≥ 10%, and were restricted to larger species (Figure 5A). In agreement with observations made using IF microscopy, staurosporine had no impact on vWf present in the culture medium over a 1 to 24 h period (Figure 5A). Remarkably, exposure of EC cultures for 24 h to purified LasB resulted in the appearance of both FnDPs and vWfDPs, the intensity and profile of which were indistinguishable from those resulting from exposure of cells to PAO1-Sec (Figure 5B). Further evidence that LasB is a major effector for degradation of ECM-associated vWf, as for Fn [29], came from exposure of EC cultures to PAO1-Sec in the presence of PA, that drastically reduced the occurrence of low M_*r*_ vWfDPs and maintained a vWf monomer profile similar to that seen with PAO1∆*lasB*-Sec (Figure 5C). Neither of the proteinase inhibitors BB-94 and Apro, nor the LPS antagonist PMB had any such inhibitory effect. Similar observations were made when evaluating FnDPs (data not illustrated). Finally, that LasB can directly target vWf was verified by exposing purified human vWf to purified LasB, which resulted in a profile of vWfDPs identical to that produced by PAO1-Sec, while PAO1∆*lasB*-Sec showed a very limited degrading activity (Figure 5D).

### Pseudomonal LasB targets membrane receptors involved in adherence and survival of endothelial cells

**Figure 6:**
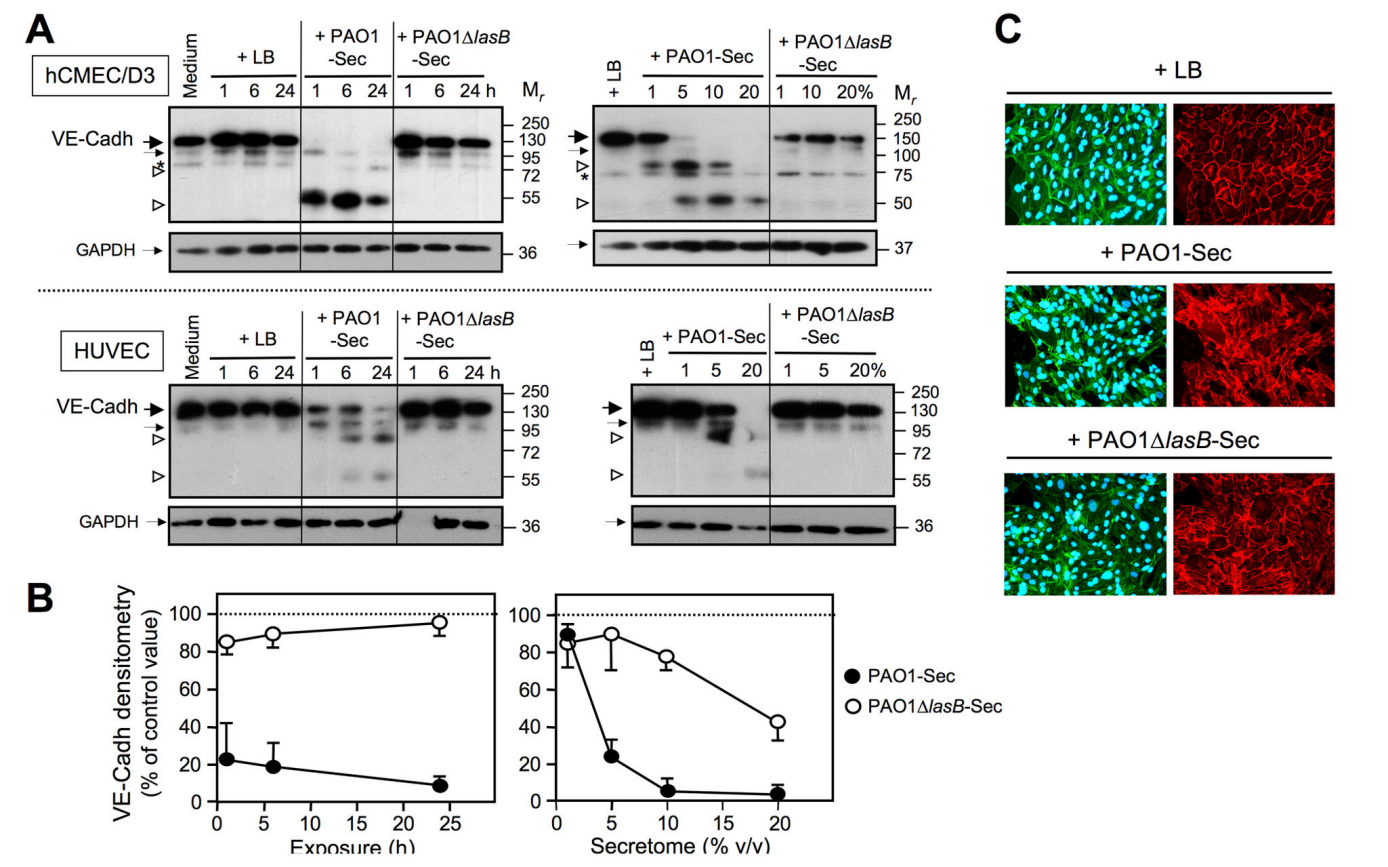
The LasB-containing secretome induces cleavage of VE-cadherin in endothelial cells in culture. (*A*) Confluent EC cultures were exposed for 1 to 24 h to low FCS culture medium alone or with 10% of either LB, PAO1-Sec, or PAO1∆*lasB*-Sec (left-hand panels), or for 24 h to culture medium with LB (20%) or with the bacterial secretomes in the range of 1 to 20% (right-hand panels). Total proteins were extracted from residual adherent cells and SDS-PAGE/IB was applied to disulfide-reduced protein extracts (5 μg *per* well) for analysis of cell-associated VE-cadherin (VE-Cadh) using an anti-VE-cadherin pAb (1 μg/ml; upper panels). Membranes were then reprobed with an anti-GAPDH mAb (0.2 μg/ml; lower panels). Portions of the films corresponding to the location of the relevant molecular species are shown with location on the left-hand side of full-length VE-cadherin (large black arrow), truncated VE-cadherin detected in control cells, and GAPDH (small black arrows), and VE-cadherin fragments generated by PAO1-Sec (open arrowheads). An asterisk indicates a non-specific interaction routinely observed with hCMEC/D3 cell extracts. Results illustrated are each representative of three or five independent experiments that were performed on HUVECs or hCMEC/D3 cells, respectively. (*B*) Densitometric analysis of bands corresponding to the full-length VE-cadherin species as illustrated in (A) is reported as the mean percentage + SEM or - SEM of the value measured for control cell extracts exposed to LB. (*C*) Confluent hCMEC/D3 cell cultures were exposed for 24 h to low FCS culture medium with 10% LB, PAO1-Sec, or PAO1∆*lasB*-Sec (Immuno). fluorescence microscopy was performed as described in the legend to Figure 5, with adherent cells being permeabilized with Triton X-100 following fixation, and before sequential labeling with the pAb against VE-cadherin (5 μg/ml, right-hand panels), then with Alexa Fluor 488-phalloidin and with DAPI (left-hand panels). Representative areas (100x magnification) are shown for one of three similar experiments performed on either hCMEC/D3 cell or HUVECs. Left-hand and right-hand panels show the same area of the same culture chamber. Primary Ab replacement by a corresponding nonspecific IgG resulted in low diffuse background labeling (not illustrated).

Besides ECM proteins, cell adhesion membrane receptors can also be targets for cleavage and shedding events accompanying the anoikis of ECs [26,45-47]. VE-cadherin (cadherin 5) is an EC-specific cadherin that is central to the integrity of endothelial adherens junctions, and thus to the organization of the endothelium as a polarized cellular barrier, and VE-cadherin plays a major role in EC survival [12,48,49]. The extracellular portion of VE-cadherin, made of five cadherin domains (EC1 to EC5), is known to be susceptible to endoproteolysis by various extracellular proteinases [50]. On SDS-PAGE/IB analysis of proteins extracted from adherent ECs, control cell extracts essentially showed the presence of the full-length membrane (m) VE-cadherin with M_*r*_ ≈ 123 kDa, along with a minor species with M_*r*_ ≈ 105 kDa that likely results from a low level of constitutive shedding of the ≈ 18 kDa intracellular domain [51] (Figure 6A). Exposure of EC cultures to 10% PAO1-Sec resulted in a rapid disappearance of full-length mVE-cadherin within 1 h (Figure 6A, left-hand panels), with a reduction of ≈ 80% as judged from a densitometric plot analysis (Figure 6B, left-hand panel), and this was accompanied by the progressive appearance of two membrane remnants with M_*r*_ ≈ 88 kDa and ≈ 59 kDa, respectively (Figure 6A). These proteolytic events could be observed for EC cultures exposed for 24 h to PAO1-Sec concentrations as low as 1 to 5%, that is, with LasB in the range of 2.5 to 12.5 nM (Figure 6A-B, right-hand panels). In contrast, exposure of ECs to 10% PAO1∆*lasB*-Sec for up to 24 h had no significant impact on mVE-cadherin (Figure 6A-B). IF microscopy analysis using the same pAb to detect cellular VE-cadherin showed, in control LB-treated cells, the expected intense labeling at the cell-to-cell boundaries (Figure 6C), corresponding to the engagement of VE-cadherin in homogeneously distributed cell junctions [34,48,49]. In cell cultures exposed to 10% PAO1-Sec for 24 h, residual adherent ECs with a flattened morphology had lost the VE-cadherin pericellular distribution although they still showed an intense labeling for the protein, likely corresponding to the proteolytic fragments seen by SDS-PAGE/IB. By contrast, ECs exposed to PAO1∆*lasB*-Sec retained most of the labeling on the cell periphery.

**Figure 7:**
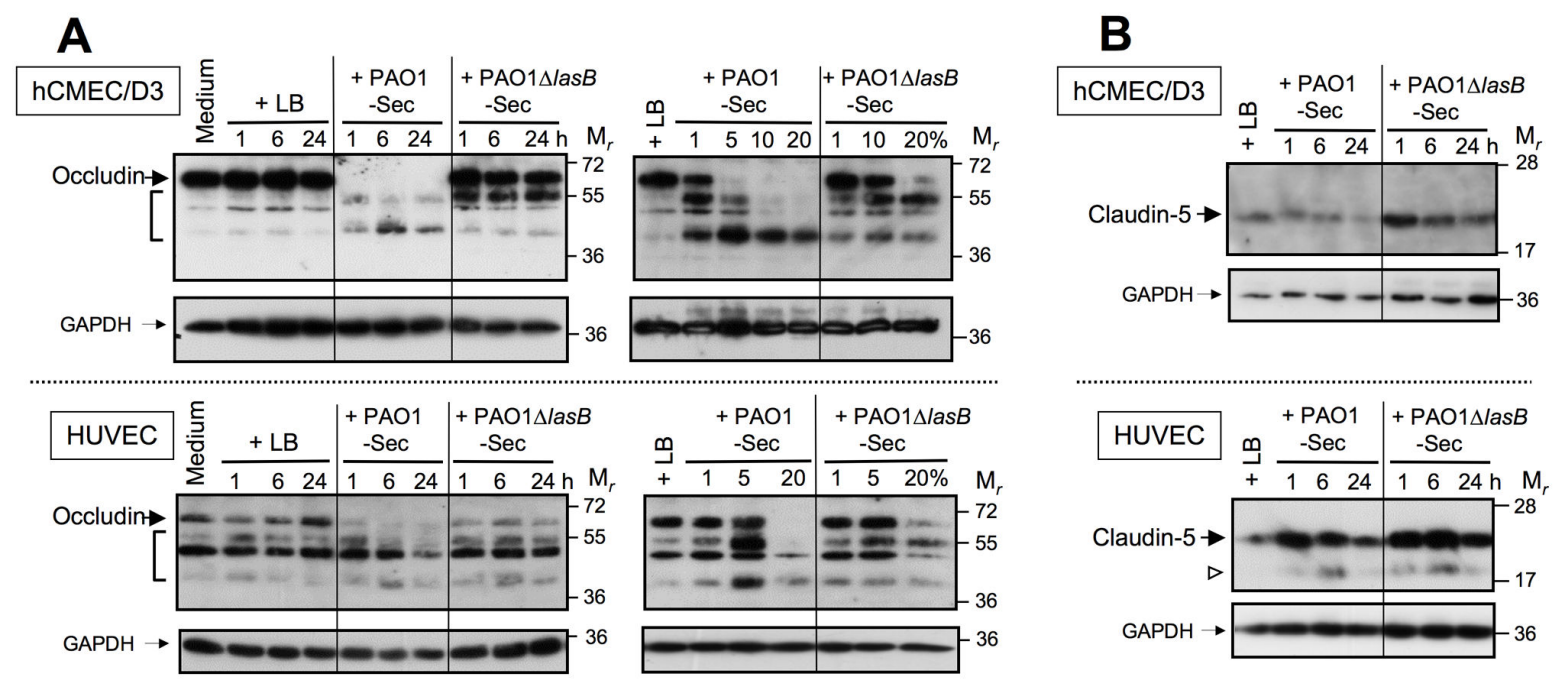
The LasB-containing secretome alters tight junction proteins in endothelial cells in culture. (*A*) Confluent EC cultures were exposed for 1 to 24 h to low FCS culture medium alone or with 10% of either LB, PAO1-Sec, or PAO1∆*lasB*-Sec (left-hand panels), or for 24 h to culture medium with LB (20%) or with the bacterial secretomes in the range of 1 to 20% (right-hand panels). Total proteins extracted from residual adherent cells were analyzed by SDS-PAGE/IB under reducing conditions (2 to 5 μg *per* well) for detection of cell-associated occludin using an anti-occludin pAb (0.5 μg/mL; upper panels), then membranes were reprobed with the anti-GAPDH mAb (lower panels). Located on the left-hand side are full-length occludin (large black arrows), GAPDH (small black arrows), and shorter species of occludin seen with bacterial secretomes (brackets in upper panels). (*B*) Confluent EC cultures were exposed for 1 to 24 h to low FCS culture medium with 10% of either LB, PAO1-Sec, or PAO1∆*lasB*-Sec, and SDS-PAGE/IB was used as above to detect cell-associated claudin-5 using an anti-claudin-5 pAb (1.25 μg/mL). Full-length claudin-5 is indicated by large black arrows, and GAPDH by small black arrows. Occasionally a previously described truncated species of claudin-5 of M_*r*_ ≈ 17 kDa [52] was detected in HUVECs, and this could be seen with either PAO1-Sec or PAO1∆*lasB*-Sec (open arrowhead in upper panels).

The endothelial tight junction membrane proteins occludin and claudins [12,34,49] are also susceptible to endoproteolysis within their extracellular domains, particularly by metalloproteinases [47,52,53]. SDS-PAGE/IB analysis similar as that performed for VE-cadherin revealed an early and complete disappearance of intact occludin (M_*r*_ ≈ 63 kDa) from EC cultures exposed to 10% PAO1-Sec for a period as short as 1 h, or to 1 to 5% secretome for 24 h, that was accompanied by the occurrence of cell-associated fragments with M_*r*_ in the range of ≈ 47 to 57 kDa (Figure 7A). By contrast, ECs exposed to 10% for PAO1∆*lasB*-Sec for up to 24 h always retained appreciable amounts of intact occludin, even though the truncated forms were also clearly detected in these cell extracts (Figure 7A). On the other hand, claudin-5, with M_*r*_ ≈ 20 kDa, showed a noticeable resistance to degradation in ECs exposed to either of the bacterial secretomes, although signal intensity tended to decrease upon exposure of ECs to secretomes for 24 h (Figure 7B).

**Figure 8:**
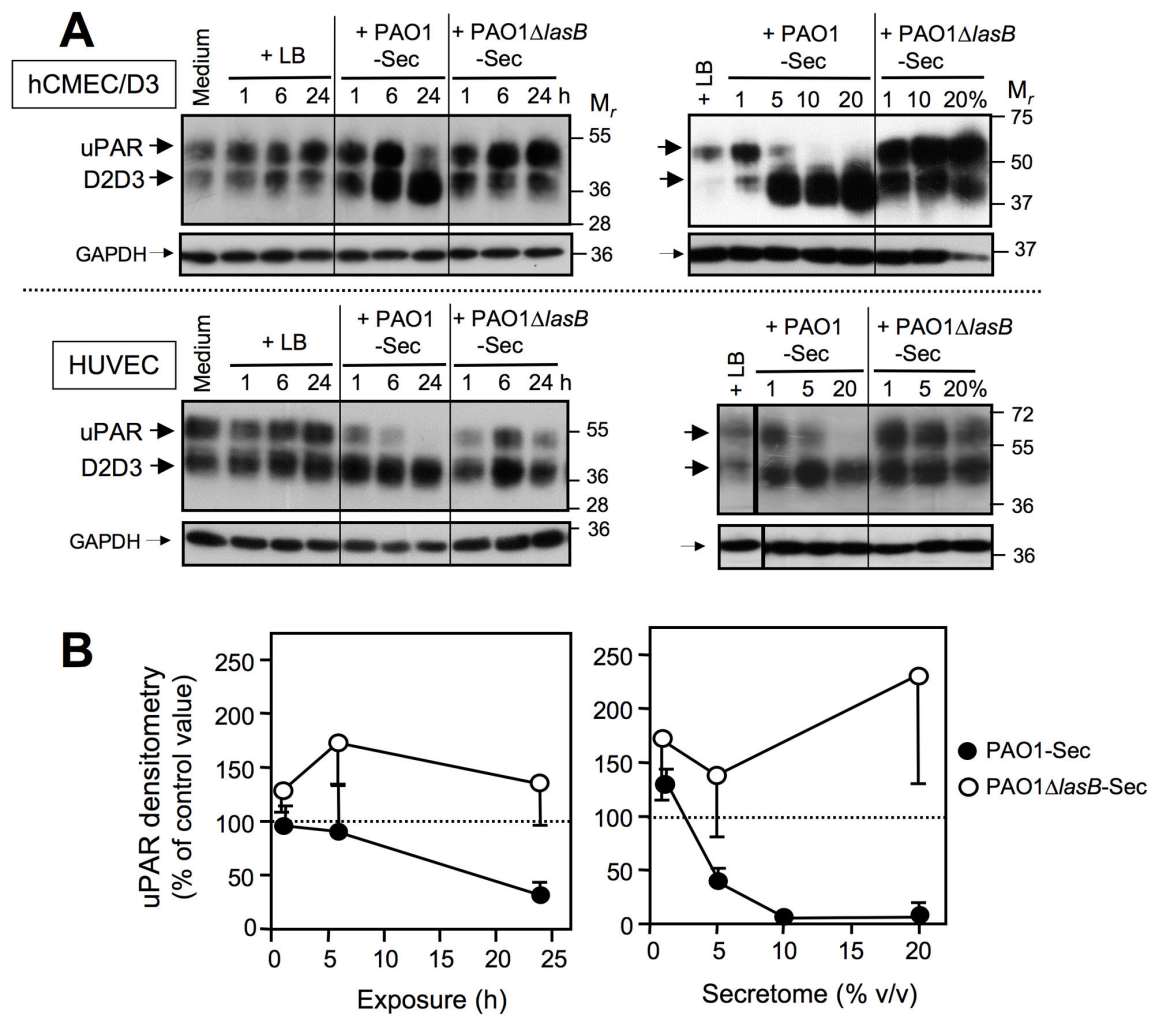
The LasB-containing secretome induces cleavage of the urokinase receptor. Confluent cultures of hCMEC/D3 cells were exposed for 1 to 24 h to low FCS culture medium alone or with 10% of either LB, PAO1-Sec, or PAO1∆*lasB*-Sec (left-hand panels in A and B), or for 24 h to culture medium with LB (20%) or with the bacterial secretomes in the range of 1 to 20% (right-hand panels in A and B). (*A*) Total proteins extracted from residual adherent cells were analyzed by SDS-PAGE/IB (5 μg *per* well) for detection of cell-associated uPAR using an anti-uPAR mAb directed against uPAR domain D2 (1/250 dilution of hybridoma cell culture medium; upper panels), then reprobed with the anti-GAPDH mAb (lower panels). Located on the left-hand side are full-length three-domain (D1D2D3) uPAR and its truncated two-domain D2D3 derivative (large black arrows), and GAPDH (small black arrows). Results illustrated are representative of four (left-hand panels) to five (right-hand panels) independent experiments performed on HUVECs or hCMEC/D3 cells. (*B*) Densitometric analysis of bands corresponding to the full-length uPAR species as illustrated in (A) is reported as the mean percentage + or - SEM of the value measured for control cell extracts exposed to LB.

Integrins, together with their physical and functional membrane partner, uPAR, are major determinants of EC attachment to subendothelial ECM adhesive proteins such as Vn, Fn, vWf and collagens, and are thus essential promoters of EC survival [5,35,54-60]. Because these receptors can be functionally regulated through cleavage by extracellular proteinases, particularly during processes of cell detachment and death [26,29,56,61], uPAR and the major EC integrins α_2_β_1_ (a receptor for Coll.I), α_5_β_1_ (a receptor for Fn), and α_V_β_3_ (a receptor for Vn and vWf) [54], were evaluated as potential LasB targets. As previously reported [21,29,38], this membrane receptor appeared in control EC extracts as a mixture of a full-length, three-domain (D1D2D3) species with M_*r*_ ≈ 57 kDa, and a truncated two-domain (D2D3) species with M_*r*_ ≈ 42 kDa that is devoid of the amino terminal D1 domain (Figure 8A). Exposure of EC cultures for 6 to 24 h to ≥ 5% PAO1-Sec progressively resulted in disappearance of full-length uPAR (Figure 8A-B), while maintaining or increasing the amounts of the cell-associated truncated D2D3 form. Conversely, exposure of EC cultures to PAO1∆*lasB*-Sec for up to 20% had no effect on the three-domain membrane uPAR (Figure 8A-B). Interestingly, a transient increase in overall uPAR quantity could be observed at low concentrations (i.e. 1% for 24 h), or after a short exposure (i.e. 6 h at 10%) to PAO1-Sec, particularly in hCMEC/D3 cells, a feature that was also obvious for ECs exposed to PAO1∆*lasB*-Sec (Figure 8A-B). As far as integrins are concerned, neither of the bacterial secretomes had any impact on the level of expression or the size of subunits α_2_, α_5_, α_V_, or β_3_, whereas some alteration of the β_1_ subunit was detected, that was however not dependent on the presence of LasB and thus had no relationship with EC detachment and death (Figure S4 in File S1).

## Discussion

*P. aeruginosa* is an important opportunistic bacterium, inducing focal and systemic vascular diseases [11,13,14]. It provides an example of how hematotropic bacteria can disrupt the vascular endothelium in order to invade host tissues [9-12], since it has been shown to interact with ECs in culture and to induce their retraction, rounding, detachment and apoptosis. Virulence factors identified so far in this process are adhesins and invasins allowing bacterial binding to ECs and their internalization as well as the type III secretion system allowing injection of pro-apoptotic effectors into host cells [13,15,17,28,62,63]. However, *P. aeruginosa* produces extracellular proteinases that are also virulence factors [15,16,19,20,24], and can be suspected to play a role in alterations of the endothelium. Indeed, (i) they are deleterious for adherence of various human cell types, including epithelial and vascular mesenchymal cells, but also HUVECs [25,29,31]; (ii) several reports suggest that virulence factors other than adhesins/invasins and intracellular toxins are implicated in EC apoptosis, and interestingly, high LasB producer strain PAO1 appears to be more pro-apoptotic than strains secreting little or no proteinases [28,62]; and (iii) *P. aeruginosa* isolates from bacteraemic patients are particularly prone to express these proteinases [22,23]. There was thus an obvious interest in evaluating the activity on ECs of these bacterial enzymes *per se*.

We establish here that, among the extracellular secreted pseudomonal toxins, LasB can severely affect the adherence and thus the survival of human ECs in culture. Massive changes in cell morphology and loss of attachment seen within 24 hours of exposure of EC cultures to PAO1-Sec, but not to PAO1∆*lasB*-Sec, are similar to those previously described for endothelial anoikis induced by purified proteinases that can be secreted by bacteria or leukocytes [26,44,64]. The LasB concentrations provided to cell cultures by the diluted secretomes we have used in the present study (i.e., approximately 15-60 nM LasB for a range of 5-20% of secretomes), are in fair agreement with those described in infected human tissues that can reach ≈ 150 nM [65]. By contrast, under similar conditions PAO1∆*lasB*-Sec induced discrete changes in cell morphology and junctions, mostly a cell elongation with only little, if any, cell detachment. A central role for LasB is further strongly suggested by the use of specific proteinase inhibitors, showing that the LasB inhibitor, PA [20], drastically reduced the cellular effects of LasB-containing secretomes, limiting them to those seen with the LasB-deficient PAO1∆*lasB*-Sec. Importantly, this observation was also verified for two LasB-containing secretomes derived from clinical bacterial isolates, thus excluding a strain-specific effect unique to PAO1. Additionally, we ruled out that that LasB acts indirectly *via* activation of pro-MMP-2 and/or pro-urokinase (and then plasminogen), two zymogens constitutively produced by ECs in culture [65] and activated by the pseudomonal proteinases [31,32], and that can *per se* induce EC detachment and death [3,4]. Strong implication of LasB in the process described here finally also relies on the use of the purified metalloproteinase that reproduced many effects noted with LasB-containing secretomes, including cell retraction and detachment, in agreement with previous observations [25]. It must be noted that multiplicity of infection (MOI) values corresponding to final dilutions of secretomes, calculated from the number of bacteria used to produce secretomes and the number of confluent ECs in culture plate wells, varied from 5 (1% secretome) to 100 (20% secretome). This is well in the range of MOI reported in studies using the PAO1 strain to investigate the implication of injected intracellular toxins in apoptosis of cultured HUVECs [66], and thus excludes any overestimation of the LasB effect as compared to that of other pseudomonal toxins.

An important feature is that residual adherent ECs remaining over a 24-hour exposure to a LasB-containing secretome do not exhibit an apoptotic phenotype and appear fully viable, a feature that corresponds to anoikis, with detachment of ECs prior to cell death [5,8], contrasting with cell cultures exposed to staurosporine that showed evidence for adherent EC apoptotic death [36]. This is in agreement with what we previously observed on human mesenchymal vascular smooth muscle cells and myofibroblasts, although detached ECs appeared to rapidly loose their viability, by contrast with detached mesenchymal cells which were more resistant to death [29]. It is indeed a common observation that ECs are more susceptible to anoikis than other monolayered polarized cells such as epithelial cells, or than fibroblasts [5,8,35,67].

Proteinase-induced vascular cell anoikis has generally been assumed to result mostly from matrilysis leading to the loss of cell anchorage and/or to the release of ECM degradation products with anti-adhesive and thus, pro-apoptotic activities [4-8,35,44]. Previous studies have shown that LasB, and to a much lesser extent AprA, can degrade the basement membrane components Coll. IV and laminin, as well as Coll. III, but scarcely Coll.I [16,29]. LasB is also highly efficient in degrading the adhesive proteins Fn and Vn known to be present in the subendothelial ECM [29, and this study]. We now further demonstrate that LasB within pseudomonal secretomes can also efficiently and rapidly (i.e., within 1 to 6 hours) degrade the subendothelial matrix-specific protein, vWf, while degradation was minimal with PAO1∆*lasB*-Sec for up to 24 h. Because the massive apoptosis induced by staurosporine was not accompanied by degradation of ECM-associated Fn and vWf, one can assume that matrilysis is not the mere consequence of proteinases released by dying cells, but rather a LasB-specific process. Interestingly, cleavage of plasma vWf by bacterial metalloproteinases was previously previously reported to be associated with anti-haemostatic and pro-haemorrhagic properties of these enzymes *in vivo* [68], and it is thus tempting to speculate that the capacity of LasB to erase the subendothelial vWf may participate in the dysregulation of haemostasis during bloodstream infections by *P. aeruginosa* [25].

A salient finding of this investigation is that an efficient cleavage of adhesion cell receptors appears to combine with matrilysis to promote EC anoikis. One striking feature is the early and extensive cleavage of two major components of endothelial cell-to-cell junctions, namely VE-cadherin at adherens junctions and occludin at tight junctions. Many inflammatory proteinases, such as neutrophil serine-proteinases and endothelial metalloproteinases, cleave the extracellular domains of VE-cadherin, possibly to facilitate transendothelial emigration of circulating leukocytes *via* interendothelial gap formation [49,50,69]. Cleavage of VE-cadherin is also a feature of a number of endothelium-interacting bacterial pathogens, and has been related to EC apoptosis [26,70]. However, in these situations, cleavage of cell-associated VE-cadherin appeared to be rather slow and incomplete [26], as opposed to our present data showing that intact membrane VE-cadherin disappears quite instantly (i.e., within one hour) upon exposure to LasB-containing secretomes. That VE-cadherin is a direct target for LasB was supported by our observation that an IgG Fc-VE-cadherin recombinant chimeric protein, encompassing the five extracellular cadherin domains, was extensively cleaved both by the LasB-containing secretome and by purified LasB, but only minimally by the LasB-deficient secretome (unpublished data). The size of the truncated membrane VE-cadherin species generated by LasB indicates that they lack the amino terminal cadherin domains implicated in the interendothelial homophilic interactions [69], and their removal could thus be a major trigger for the disruption of cell monolayers. Not only is VE-cadherin a central molecular organizer of initial adherens junctions in confluent ECs, it is also implicated in the subsequent organization of tight junctions [49]. It is thus interesting that the early extensive LasB-dependent cleavage of VE-cadherin was accompanied by a similar event targeting one major component of tight junctions, occludin. The cleavage of occludin and occurrence of cell-associated fragments with different sizes as observed here, have been previously reported upon exposure of cells to metalloproteinases, and this cleavage has often been noted to be concomitant with epithelial or endothelial cell apoptosis [47,52,53]. Finally, although several studies have shown that MMPs can proteolyse various claudins, including claudin-5, along with occludin [52,53], it is noteworthy that endothelial claudin-5 appeared rather insensitive to LasB.

Integrins α_2_β_1,_ α_5_β_1_ and α_V_β_3_ are receptors among those most implicated in EC attachment to ECM components such as collagens, Fn, or Vn and vWf, respectively, providing cells with major survival, and particularly anti-anoikis, signaling pathways [5,35,54-57,67]. Interestingly, the proteolytic processing of integrin subunits α_5_ and/or β_1_ has been reported to accompany cell detachment and death, including that induced by bacterial proteinases [26,46,61]. The present study shows, however, that endothelial anoikis induced by LasB-containing secretomes was not specifically associated with structural alterations of any of these integrins, as previously noted with human vascular mesenchymal cells [29]. In contrast, the integrin-associated uPAR on ECs was subject to a LasB-dependent progressive shedding of its N-terminal D1 domain, leaving a truncated D2D3 membrane form of the receptor. This discrete proteolytic event is a significant potential trigger for cell detachment and death. Indeed, uPAR is an important promoter and regulator of cell adhesion processes, particularly in ECs, *via* its intrinsic capacity to bind Vn, and as an integrin-associated protein that stimulates cell adherence, migration, and survival [55-58]. uPA-uPAR complexes also participate in anti-apoptotic signaling pathways in both epithelial cells and ECs, allowing a sustained expression of Bcl-xL or the X-linked inhibitor of apoptosis, respectively [58-60]. Since in all of these processes, engagement of uPAR by uPA appears mandatory, the proteolytic removal of the D1 domain from the cell surface, that disrupts uPA/uPAR interactions, may thus be a major pathway for the downregulation of uPAR-associated functions promoting cell adherence and survival [38,56].

Taken together, our present results indicate that rapid and extensive, LasB-triggered cleavage of junctional proteins such as VE-cadherin and occludin, accompanied by that of uPAR and of major ECM components, would undoubtedly alter cell adherence and survival, and engage ECs in anoikis. It is however obvious that the pseudomonal secretome contains substance(s), that, in addition to LasB, can alter the morphology of confluent ECs by acting on intercellular junctions. LasB-deficient secretomes still contain proteinases such as AprA and PrpL, but because they induce very limited proteolytic events within the ECM and adhesion receptors, it can be assumed that these proteinases have low, if any, activity in this context. This assumption also applies to LPS, a cell wall component released in the secretomes from Gram-negative bacteria, and a potent virulence factor particularly active on the endothelium [9,15], but for which we have excluded a major role in the process described here for ECs, as did others [28]. Other pseudomonal secreted toxins may be considered as potential candidates to modulate endothelial permeability, including the siderophoric pigments, and/or surfactant-like rhamnolipids [15,71]. In addition, most *P. aeruginosa* strains can secrete, under particular microenvironmental conditions, a phospholipase/sphingomyelinase complex that was recently shown to induce EC cytotoxicity and to have anti-angiogenic activity [72]. These molecules were not explored in our present study since they are not produced in our bacterial culture conditions (unpublished data), but clearly deserve future investigation as putative anoikis-inducing factors. Furthermore, by using acellular secretomes we do not take into account the type III secretion system-dependent pseudomonal toxins such as ExoS/ExoT, ExoU and ExoY that are injected into host cells by living bacteria [15,17,62,63]. By acting on the cytoskeleton and promoting cell rounding and detachment, these effectors would probably act synergistically with LasB, as previously demonstrated for an other inflammatory cell process, i.e. wound healing [31,73].

In conclusion, although the *P. aeruginosa* extracellular proteasome is one of the most extensively studied for interaction with host proteins and cells [15,16,19,20,24,29,31,33], there has been so far little or no analysis of its impact on ECs [25]. Our present study provides new insight into how secreted bacterial proteinases affect the endothelial barrier, and more particularly pinpoints the central role of LasB and the diversity of its targets. This enzyme thus can be regarded as a prototype for the analysis of haemorrhagic, thermolysin-like metalloproteinases, a class of proteinases that are receiving a growing attention as bacterial pathogenic factors [15,68].

## Supporting Information

File S1 **This file contains Protocol S1 and Figure S1 through Figure S4.** Protocol **S1**, Preparation of extracellular bacterial secretomes and proteinase activity assays. Figure S1, Profile of proteinases in the secretome of wild type and LasB-deficient strains of *P*. *aeruginosa*. Figure S2, Clinical pseudomonal secretomes drastically alter adherence of human endothelial cells in culture in a LasB-dependent fashion. Figure S3, Residual adherent endothelial cells in the presence of pseudomonal secretomes are fully viable. Figure S4, The LasB-containing secretome does not proteolytically alter the major endothelial integrins.
(DOC)
