# Supplementary material for: Disruption of the Endothelial Barrier by Proteases from the Bacterial Pathogen Pseudomonas aeruginosa: Implication of Matrilysis and Receptor Cleavage
Source: PLoS One. 2013 Sep 19;8(9):e75708. doi: 10.1371/journal.pone.0075708 (PMC3777978; doi:10.1371/journal.pone.0075708)
Supplement: File S1 — This file contains Protocol S1 and Figure S1 through Figure S4. Protocol S1, Preparation of extracellular bacterial secretomes and proteinase activity assays. Figure S1, Profile of proteinases in the secretome of wild type and LasB-deficient strains of P. aeruginosa. Figure S2, Clinical pseudomonal secretomes drastically alter adherence of human endothelial cells in culture in a LasB-dependent fashion. Figure S3, Residual adherent endothelial cells in the presence of pseudomonal secretomes are fully viable. Figure S4, The LasB-containing secretome does not proteolytically alter the major endothelial integrins. (DOC) [file pone.0075708.s001.doc]

**Disruption of the endothelial barrier by proteases from the bacterial pathogen *Pseudomonas aeruginosa*: implication of matrilysis and receptor cleavage**

Nathalie Beaufort, Elisabeth Corvazier, Saouda Mlanaoindrou, Sophie de Bentzmann, Dominique Pidard

**Protocol S1. Preparation of extracellular bacterial secretomes and proteinase activity assays**

For preparation of the bacterial secretomes (abbreviated as Sec), bacteria were grown overnight at 37°C with shaking in Luria-Bertani broth (LB) liquid medium (Invitrogen Corp., Camarillo, CA). Sterile, bacterium-free culture supernatants were then obtained by centrifugations at 6,000 *g* and 12,000 *g*, both for 10 min, and further filtration through 0.22 μm membranes, before being stored in aliquots at -80°C. Experimental dilutions of secretomes or LB control medium are expressed as the percentage of the final volume of culture medium added to endothelial cells (ECs). Multiplicity of infection (MOI) values corresponding to final dilutions of secretomes were calculated from the number of bacteria used to produce secretomes and the number of confluent ECs in culture plate wells. Secretomes from the previously characterized LasB-producing *Pseudomonas aeruginosa* strain PAO1 and its LasB-deficient isogenic mutant PAO1∆*lasB* [1-3] were mainly used in this study. Psa1 and Psa2 are two different, LasB-producing *P. aeruginosa* clinical isolates derived from patients with infective endocarditis subsequent to implantation of a pacemaker. Their secretomes were used in selected experiments, and have been extensively described before [4].

Proteinases in bacterial secretomes and purified LasB preparations (specific activity, 260 U/mg; Elastin Products Company, Owensville, MO) were analyzed by sodium dodecyl sulfate-polyacrylamide gel electrophoresis (SDS-PAGE) coupled to zymography on 7.5% polyacrylamide gels containing 0.2% (w/v) bovine gelatin (Gibco-Invitrogen) followed by Coomassie blue staining, as previously described [3]. The LasB-dependent elastolytic activity of the pseudomonal secretomes was measured using elastin-Congo red (Sigma-Alrich, Saint-Louis, MO) as substrate, with purified LasB in the range of 3 to 25 nM for calibration, as previously described [4,5]. LasB concentration was in the range of 250 to 300 nM in PAO1-Sec preparations, it was about two-fold higher in Psa1-Sec and Psa2-Sec (455 nM and 465 nM, respectively), while it was undetectable in PAO1∆*lasB*-Sec [4].

**Figure S1. Profile of proteinases in the secretome of wild type and LasB-deficient strains of *P. aeruginosa*.** The enzymatic profile of the two sets of secretomes obtained from the pseudomonal reference strain PAO1 and its isogenic mutant PAO1∆*lasB* (*PAO1-Sec* and *PAO1∆*lasB*-Sec*, respectively, 5 ml *per* well; right-hand panel), together with the two purified LasB preparations used in this study (0.5 μg protein *per* well; left-hand panel), all noted #1 and #2, was assessed by SDS-PAGE/zymography. Positions of M*_r_* calibration standard proteins are indicated on the left-hand side of gels, while those of pseudomonal proteinases are indicated by arrows on the right-hand side. Illustrated are scanned images representative of one to three similar analyses. Note that PAO1∆*lasB*-Sec contains levels of the metalloproteinase AprA and serine-proteinase PrpL that are similar to those found in PAO1-Sec, but no LasB activity, while purified LasB preparations are devoid of the other pseudomonal proteinases.


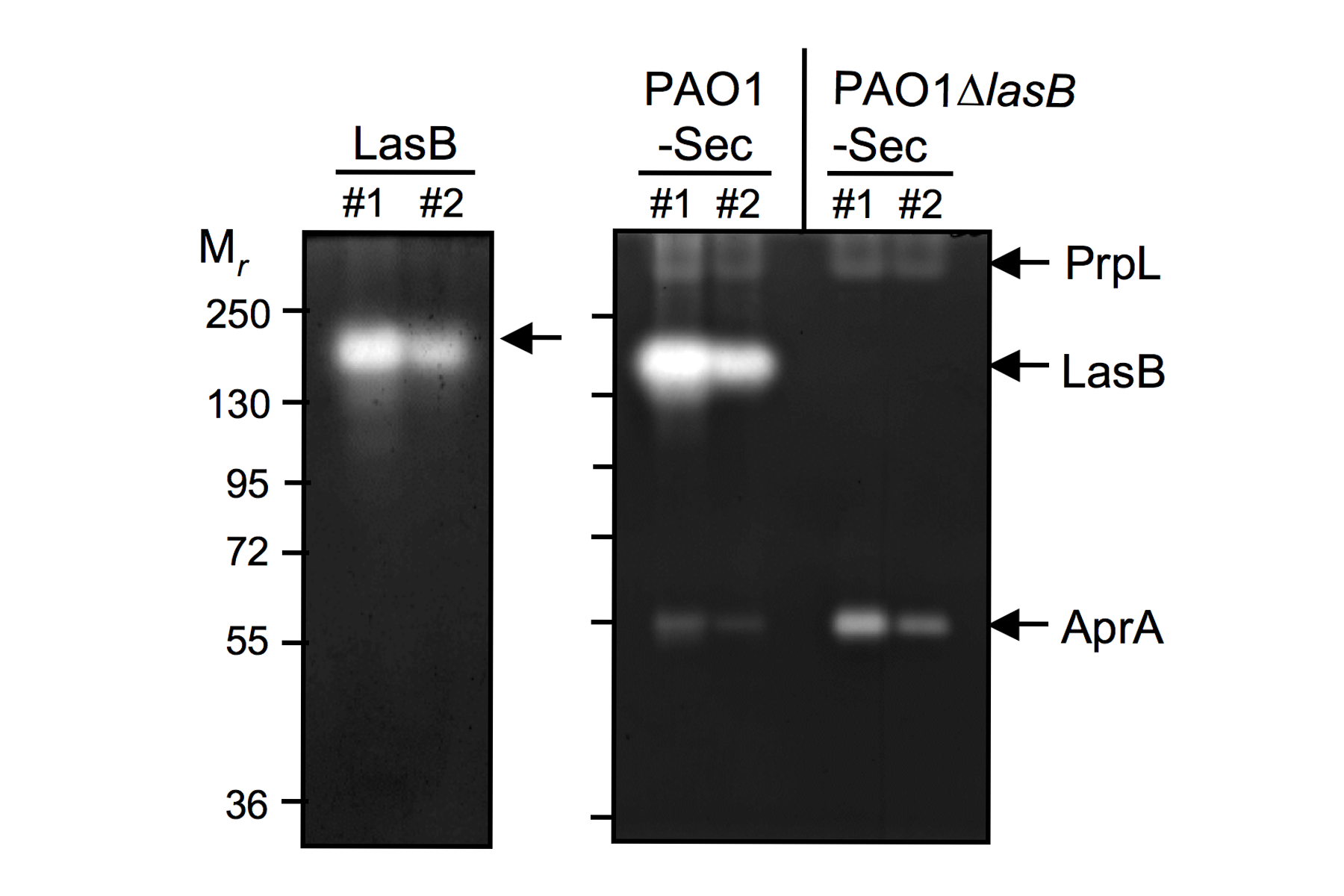


**Figure S2. Clinical pseudomonal secretomes drastically alter adherence of human endothelial cells in culture in a LasB-dependent fashion.** Confluent HUVEC cultures were exposed for 24 h to low FCS (0.2%) culture medium alone or either with LB, PAO1∆*lasB*-Sec or PAO1-Sec (10%, *i.e.* about 25 nM LasB for the latter secretome), or with Psa1-Sec or Psa2-Sec (5%, *i.e.* about 23 nM LasB), in the absence (grey bars) or presence (black bars) of phosphoramidon (50 μM; *PA*). The quantity of residual adherent cells was then assessed using the CV staining assay in parallel triplicate wells treated identically. Results are expressed as the mean OD value measured at 590 nm in triplicate wells after subtraction of the value at 620 nm (OD_590-620_), and are the means + SEM of three independent experiments. Note that in this series of experiments, cell density in PAO1∆*lasB*-Sec-treated wells was not significantly (*P* > 0.4; *NS*) decreased as compared to control culture wells (*+ LB*; solid line), whereas it was significantly decreased (* *P* ≤ 0.01) by ≈ 30% in PAO1-Sec-treated wells, and by ≈ 90% in Psa1-Sec- and Psa2-Sec-treated cultures. When the LasB inhibitor PA was present, cell detachment induced by LasB-containing secretomes was largely blocked, with the quantity of residual adherent cells being not statistically different from that measured in PAO1∆*lasB*-Sec-treated wells (*P* ≥ 0.09; *NS*, dashed line), except with Psa1-Sec (** *P* ≤ 0.01).


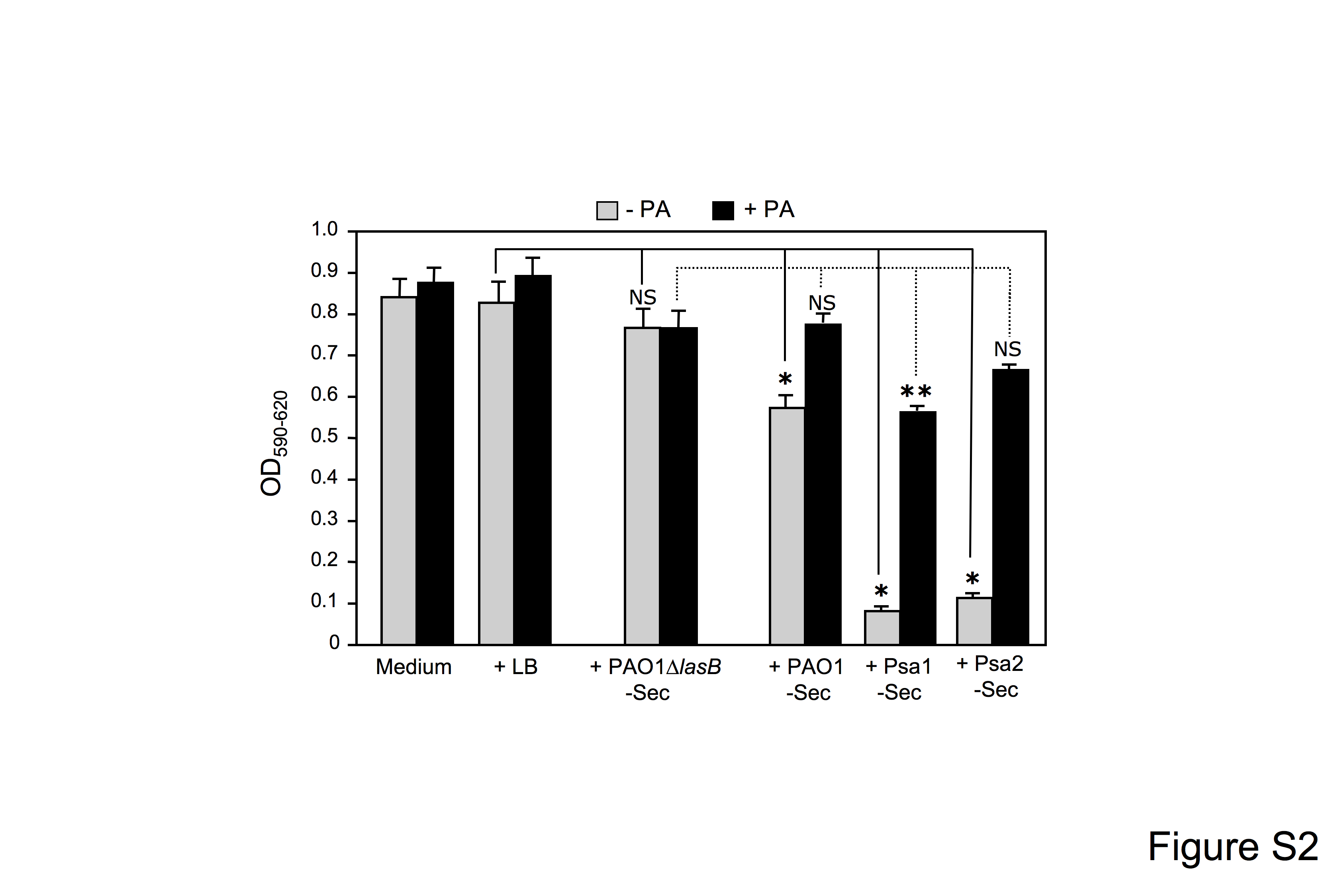


**Figure S3. Residual adherent endothelial cells in the presence of pseudomonal secretomes are fully viable**. Confluent EC cultures were exposed for 24 h to low FCS (0.2%) culture medium alone or with either 20% LB or PAO1∆*lasB*-Sec, or to 5 or 20% PAO1-Sec, or for 4 h to 5 μM staurosporine. The quantity of residual adherent cells was then assessed using the CV staining assay, whereas their viability was evaluated using the MTT assay in parallel triplicate wells treated identically. Results are expressed as the mean OD value measured at 590 nm in triplicate wells after subtraction of the value at 620 nm (OD_590-620_), and are the means + SEM of three independent experiments (hCMEC/D3 cells), or means + range of two independent experiments (HUVECs). The MTT / CV ratio calculated for each experimental condition as an index of cell survival is indicated on top of the histograms. Note that this ratio did not differ statistically between hCMEC/D3 cell cultures exposed to bacterial secretomes as compared to culture medium with LB (*P* ≥ 0.4).

**
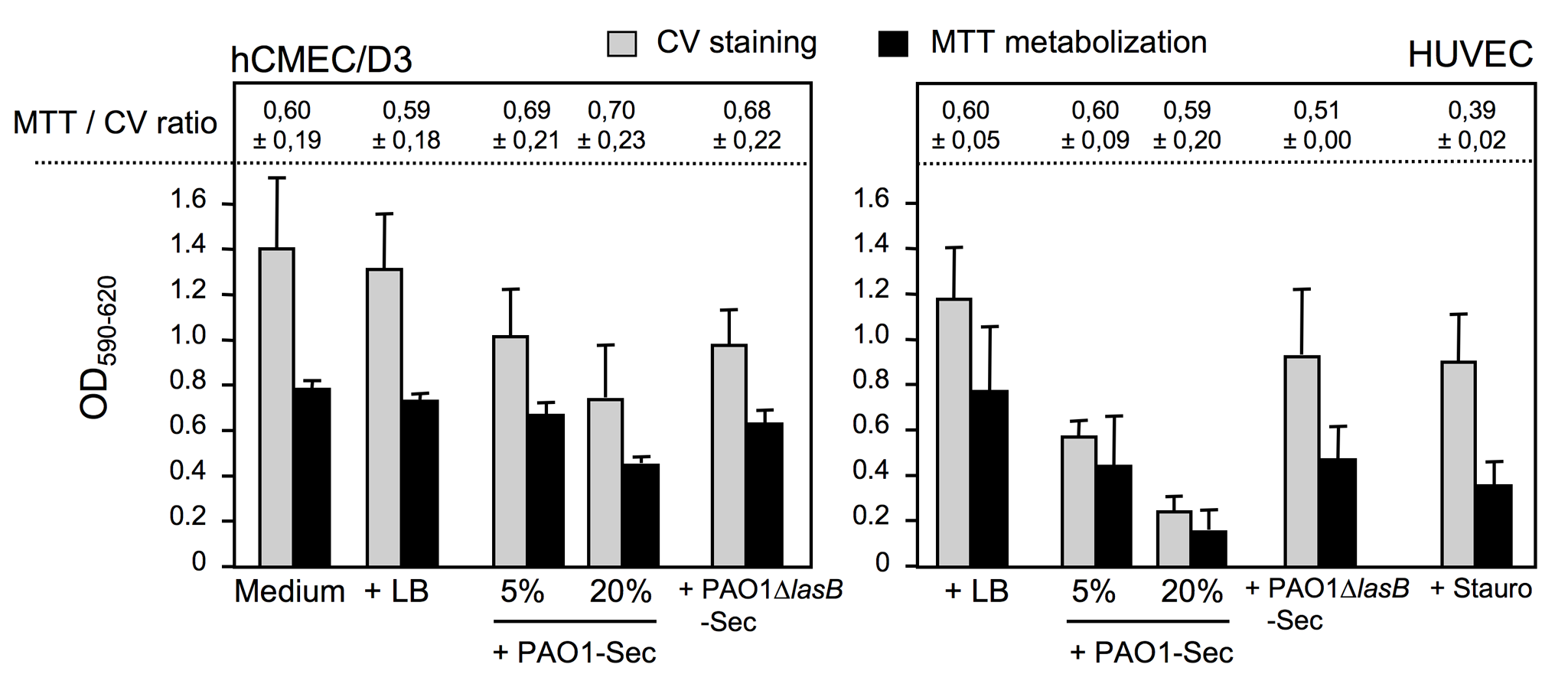
**

**Figure S4. The LasB-containing secretome does not proteolytically alter the major endothelial integrins.** Confluent HUVECs were exposed for 1 to 24 h to low FCS culture medium with 10% of either LB, PAO1-Sec, or PAO1∆*lasB*-Sec. Proteins extracted from residual adherent cells were analyzed by immunoblotting (IB) coupled to SDS-PAGE (5 μg *per* well) for integrity of the major EC integrins α_2_β_1_ (a receptor for collagen type I), α_5_β_1_ (a receptor for fibronectin), and α_V_β_3_ (a receptor for vitronectin and von Willebrand factor), *via* detection of integrin subunits β_1_, α_2_, and α_5_ (disulfides reduced), and α_V_ and β_3_ (unreduced), using polyclonal antibodies (pAbs) directed against (from top to bottom) β_1_ (0.8 μg/ml), β_3_ (1/1000 dilution), α_V_ (1/200 dilution), α_2_ (0.4 μg/ml), or α_5_ (0.4 μg/ml). pAbs H293, H104 and M106 to α_2_, α_5_ and β_1_, respectively, were from Santa Cruz Biotechnology (Santa Cruz, CA), pAb AB1930 to α_V_, from Millipore (Billerica, MA), and the non-commercial pAb against β_3_ has been previously described [6]. Monoclonal antibody 2D4A7 to glyceraldehyde-3-phosphate dehydrogenase (GAPDH), was from Abcam (Cambridge, UK). On the left-hand side are located full-length integrin subunits (large black arrows), as well as GAPDH as loading protein control (small black arrow). Results illustrated are representative of two to three independent experiments performed on HUVECs or hCMEC/D3 cells. Note that none of the integrin subunits b_3_ (M*_r_* ≈ 99 kDa), α_V_ (large chain, M*_r_* ≈ 134 kDa), α_2_ (M*_r_* ≈ 133 kDa), or α_5L_ (large chain, M*_r_* ≈ 126 kDa) showed any proteolytic events. A progressive shift of subunit β_1_ (M*_r_* ≈ 126 kDa) to a shorter species (M*_r_* ≈ 114 kDa; small black arrow) was observed when cells were exposed to either the LasB-containing or the LasB-decicient secretome, with no relationship with cell detachment.


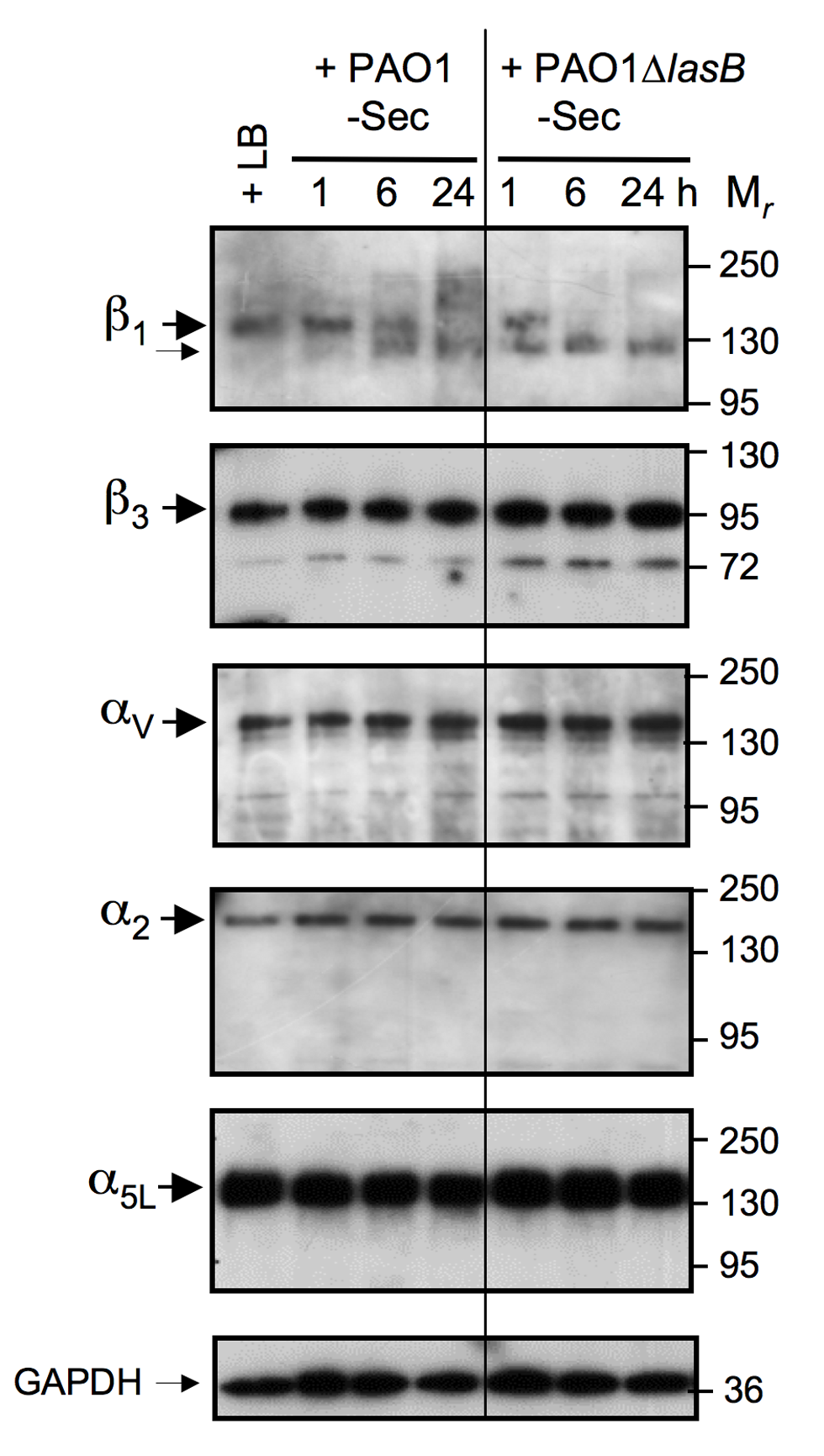


**REFERENCES**

1. McIver KS, Kessler E, Olson JC, Ohman D (1995) The elastase propeptide functions as an intramolecular chaperone required for elastase activity and secretion in *Pseudomonas aeruginosa*. Mol Microbiol 18: 877-889.

2. de Bentzmann S, Polette M, Zahm JM, Hinnrasky J, Kileztky C, *et al.* (2000) *Pseudomonas aeruginosa* virulence factors delay airway epithelial wound repair by altering the actin cytoskeleton and inducing overactivation of epithelial matrix metalloproteinase-2. Lab Invest 80: 209-219.

3. Beaufort N, Seweryn P, de Bentzmann S, Tang A, Kellermann J, *et al.* (2010) Activation of human pro-urokinase by unrelated proteases secreted by *Pseudomonas aeruginosa*. Biochem J 428: 473-482.

4. Beaufort N, Corvazier E, Hervieu A, Choqueux C, Dussiot M, *et al.* (2011) The thermolysin-like metalloproteinase and virulence factor LasB from pathogenic *Pseudomonas aeruginosa* induces anoikis of human vascular cells. Cell Microbiol 13: 1149-1167.

5. Caballero AR, Moreau JM, Engel LS, Marquart ME, Hill, *et al.* (2001) *Pseudomonas aeruginosa* protease IV enzyme assays and comparison to other *Pseudomonas* proteases. Anal Biochem 290: 330-337.

6. Pidard D, Frelinger AL, Bouillot C, Nurden AT (1991) Activation of the fibrinogen receptor on human platelets exposed to alpha chymotrypsin. Relationship with a major proteolytic cleavage at the carboxyterminus of the membrane glycoprotein IIb heavy chain. Eur J Biochem 200: 437-447.
